# Polysaccharide-Based Organic-Inorganic Hybrid Carriers with Alginate as a Reference Matrix: Structure-Property Relationships and Emerging Applications in Encapsulation and Controlled Release

**DOI:** 10.3390/polym18172047

**Published:** 2026-08-23

**Authors:** Agata Wawrzyńczak, Agnieszka Kłosowska, Agnieszka Feliczak-Guzik

**Affiliations:** Department of Applied Chemistry, Faculty of Chemistry, Adam Mickiewicz University in Poznań, Uniwersytetu Poznańskiego 8, 61-614 Poznań, Poland; agnieszka.klosowska@amu.edu.pl

**Keywords:** alginate, polysaccharides, organic-inorganic hybrids, hybrid polymers, encapsulation, controlled release, volatile compounds, bioactive compounds

## Abstract

Polysaccharide-based organic-inorganic hybrid carriers combine renewable polymer matrices with inorganic phases that can modify mechanical integrity, swelling, barrier performance, payload retention, and release behavior. This review critically evaluates alginate as a reference matrix together with chitosan, cellulose/nanocellulose, starch/maltodextrin, pectin, carrageenan, and related polysaccharides, focusing on how matrix chemistry, inorganic-phase properties, interfacial interactions, and fabrication route govern encapsulation efficiency, loading, structural stability, swelling, mechanical and barrier properties, storage retention, and release kinetics. Silica and mesoporous silica, clays and halloysite, layered double hydroxides (LDHs), metal oxides, hydroxyapatite, magnetic particles, and metal-organic frameworks are compared according to their reservoir, reinforcing, diffusion-controlling, responsive, and safety-related functions. Representative quantitative findings illustrate the importance of hybrid architecture; for example, incorporation of LDHs into an alginate matrix reduced erythropoietin release after 108 h from 86% to 24% while increasing mechanical performance by approximately 5–30-fold. In this review, particular attention is given to volatile and bioactive compounds, for which storage retention, oxidation stability, headspace behavior, and application-relevant release are as important as initial encapsulation efficiency. Key challenges, such as long-term stability, standardization of release studies, scalability, safety assessment, and performance in real formulations, are also discussed, together with future directions for sustainable, application-specific hybrid carrier systems. Overall, the review provides a structure-property-application framework for selecting matrix-filler-processing combinations for controlled-release systems.

## 1. Introduction

### 1.1. Organic-Inorganic Hybrid Materials

Polysaccharide-based polymers are widely used in encapsulation and delivery systems because of their biocompatibility, biodegradability, abundance, and ability to form hydrogels and structured matrices. Incorporation of inorganic components into these biopolymeric networks leads to the formation of organic-inorganic hybrid materials, in which both the polysaccharide and inorganic phases contribute to the overall properties of the system through synergistic interactions rather than acting as separate constituents. Such hybrids can exhibit enhanced mechanical strength, thermal stability, barrier properties, adsorption capacity, stimuli responsiveness, and controlled-release performance, while the distribution of the inorganic phase within the matrix strongly influences encapsulation efficiency, permeability, swelling behavior, and storage stability. In this review, the term “organic-inorganic hybrid” is reserved for materials in which a polysaccharide phase and a discrete inorganic component retained in the final carrier jointly determine material properties. Accordingly, ionotropic crosslinking with soluble multivalent ions alone is not considered sufficient to classify a system as an organic-inorganic hybrid.

Polysaccharide-based polymers are important sustainable materials because of their renewable origin, structural diversity, abundant functional groups, aqueous processability, and broad applicability. Hydroxyl, carboxyl, amino, sulfate, and acetyl groups enable hydrogen bonding, ionic interactions, covalent modification, metal-ion coordination, and interactions with inorganic surfaces, thereby influencing morphology, swelling, mechanical and barrier properties, and release behavior. These features are particularly valuable in encapsulation systems, which must protect active compounds during processing and storage while enabling controlled release. This is especially important for labile, volatile, or oxidation-sensitive compounds, including flavors, fragrances, essential oils, cosmetic actives, nutraceuticals, and other functional molecules susceptible to light, oxygen, moisture, temperature, or formulation components [1,2,3,4,5,6,7].

Alginate is widely used for encapsulation because it forms hydrogels under mild aqueous conditions through ionotropic crosslinking with multivalent cations [8,9,10], avoiding high temperatures, aggressive solvents, and harsh initiators [11]. Its carboxylate groups enable ionic complexation and interactions with inorganic phases [12], while the mannuronic-to-guluronic acid ratio affects chain stiffness, ion affinity, gel strength, porosity, and swelling. These properties support the preparation of beads, microcapsules, hydrogels, films, and composite carriers [13,14]. However, moderate mechanical strength, ion-exchange sensitivity, burst release, limited long-term stability, and dependence on pH and ionic strength have motivated the development of mixed-polysaccharide and organic-inorganic systems [15]. Other polysaccharides provide complementary properties for hybrid carriers [16]. Chitosan contributes cationic amino groups, pH responsiveness, and polyelectrolyte complexation with anionic polysaccharides [17,18]. Cellulose and nanocellulose provide mechanical reinforcement, film formation, and modifiable hydroxyl-rich surfaces [19]. Starch and maltodextrin offer low cost, good processability, and compatibility with drying-based encapsulation [20,21]. Pectin and carrageenan provide anionic gel-forming functionality, whereas xanthan gum and related polysaccharides act mainly as rheology modifiers and stabilizers [22,23]. Thus, polysaccharide selection is a key design parameter governing the structure and function of hybrid materials.

The inorganic component may provide reinforcement, adsorption sites, diffusion barriers, pH responsiveness, thermal stability, magnetic behavior, antimicrobial activity, or additional interactions with the encapsulated compound. It may be incorporated as preformed particles, generated in situ, deposited on surfaces, or introduced during gelation, drying, extrusion, coating, or layer-by-layer (LbL) assembly [24]. Its location within the matrix or at interfaces strongly influences encapsulation efficiency, swelling, permeability, release rate, and storage stability. Common inorganic components include silica and mesoporous silica, layered clays such as halloysite nanotubes and montmorillonite, layered double hydroxides, metal oxides, calcium phosphates, and magnetic nanoparticles. These phases can improve structural stability, increase diffusion-path tortuosity, provide adsorption or ion-exchange sites, enhance retention of volatile or hydrophobic compounds, or introduce antimicrobial, UV-protective, bioactive, or magnetic functions. Their performance depends not only on composition but also on particle size, surface chemistry, dispersion, interfacial adhesion, matrix compatibility, and interactions with the encapsulated compound [25,26,27].

Organic-inorganic hybrids therefore include, among others, hydrogel beads containing mesoporous silica, alginate systems with halloysite or layered double hydroxides, chitosan-silica hydrogels, polysaccharide/silica microcapsules, nanocellulose films containing mineral or metallic particles, starch- or maltodextrin-based hybrid powders, pectin composite gels, and multilayer capsules containing inorganic nanophases. These materials may take the form of beads, particles, films, coatings, aerogels, membranes, or porous scaffolds and may operate in food, cosmetic, biomedical, packaging, textile, environmental, or agricultural systems. Despite their diversity, they share a common principle: the polysaccharide phase provides processability, biodegradability, and chemical functionality, whereas the inorganic phase modifies stability, transport pathways, interfacial interactions, and release behavior [25,28,29,30,31,32,33,34].

### 1.2. Research Gaps, Novelty, and Scope

The need for a systematic review is evident from the current literature published between 2016 and 2026. Numerous reviews have discussed alginate-based encapsulation, alginate hydrogels, chitosan-alginate nanoparticles, chitosan particulate carriers, essential-oil encapsulation, polysaccharide-based films, and active packaging materials. These publications are valuable and provide detailed knowledge about individual material classes or selected applications. However, many of them remain focused on one polymer family, one type of encapsulated compound, one fabrication method, or one application sector, such as drug delivery, wound healing, food packaging, or environmental remediation. As a consequence, information on hybrid polysaccharide materials is often fragmented. The relationship between polysaccharide chemistry, inorganic phase selection, processing method, morphology, and release behavior is rarely analyzed in a unified comparative framework. This fragmentation makes it difficult to answer practical design questions, such as which polysaccharide-inorganic combination is most suitable for volatile compounds, which systems favor long-term retention, which structures reduce burst release, and which fabrication methods are most compatible with scalable industrial processing. This gap is particularly visible for carriers of volatile and bioactive compounds. Many encapsulation studies focus on drugs, dyes, proteins, or environmental contaminants, whereas flavors, fragrances, essential oils, and cosmetic active compounds pose additional challenges related to volatility, hydrophobicity, susceptibility to oxidation, and performance in complex formulations. For these compounds, encapsulation efficiency alone is not sufficient to evaluate material performance. Long-term retention during storage, protection from evaporation or oxidation, compatibility with the final product, sensory release profile, and trigger-dependent release under use conditions are equally important. Organic-inorganic polysaccharide hybrids are promising in this respect because they can combine the mild processing and biodegradability of polysaccharides with the barrier, adsorption and reinforcing effects of inorganic components. Nevertheless, systematic comparisons between different hybrid architectures remain limited.

**Figure 1:**
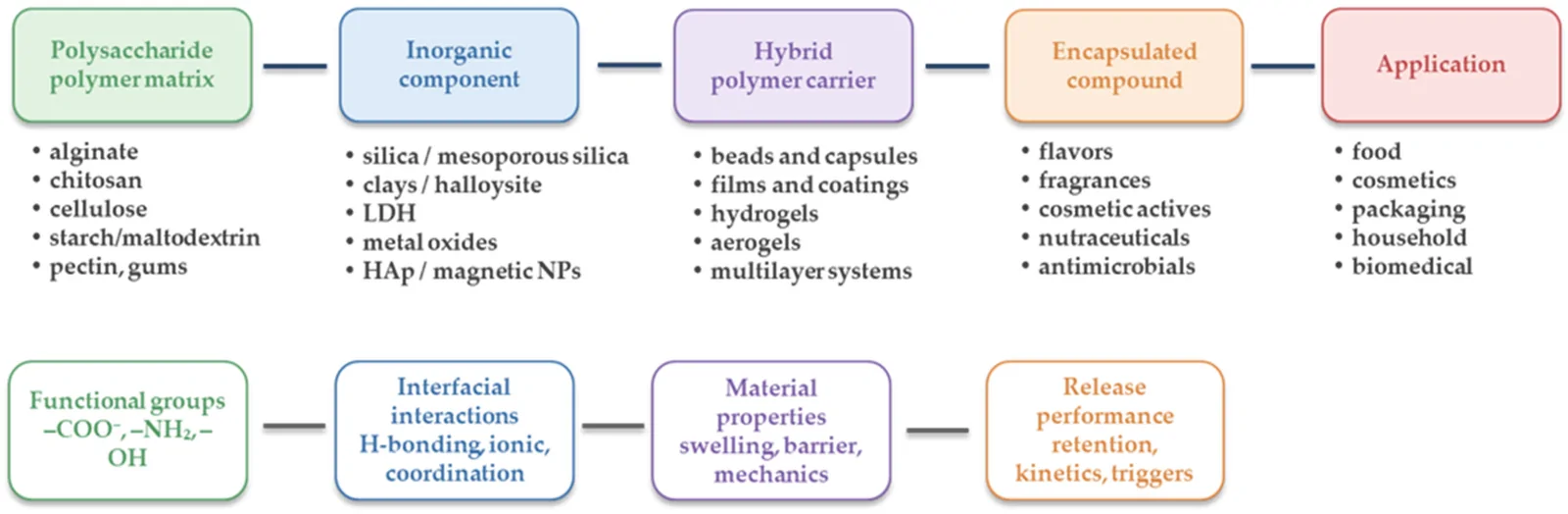
Design map for polysaccharide-based organic-inorganic hybrid polymers used as encapsulation and controlled-release systems. The scheme links the selection of the polysaccharide matrix, inorganic component, and fabrication strategy with structure-property relationships, encapsulation performance, and target applications.

The present review addresses this gap by providing a comparative, design-oriented analysis of polysaccharide-based organic-inorganic hybrid carriers within a unified structure-property-application framework. This design-oriented framework is schematically illustrated in Figure 1. Rather than considering alginate as an isolated hydrogel matrix or focusing on a single class of inorganic fillers, fabrication methods, or applications, we compare how polysaccharide chemistry, inorganic-phase characteristics, interfacial interactions, and processing routes jointly determine carrier morphology, swelling, mechanical and barrier properties, encapsulation efficiency, retention, and release kinetics. Particular emphasis is placed on volatile and bioactive compounds, including flavors, fragrances, essential oils, cosmetic actives, and nutraceuticals, for which performance de-pends not only on initial encapsulation efficiency but also on storage retention, oxidation stability, headspace behavior, and release under application-relevant conditions. Accordingly, this review aims to identify practical relationships between matrix, inorganic phase, processing strategy, payload properties, and target application, thereby providing a framework for the rational selection of hybrid carrier systems.

The scope of this review is primarily based on the literature published within the last decade, covering the period from 2016 to 2026. This time window is broad enough to include earlier composite hydrogel beads and silica-polysaccharide systems that shaped the current field, while also covering recent work on halloysite, layered double hydroxides, metal-organic frameworks, essential oil carriers, polysaccharide films, active packaging concepts, and application-ready microcapsules. However, the review does not aim to provide an exhaustive catalogue of all polysaccharide-based composites, but rather to organize representative examples according to material design principles. Emphasis is placed on systems in which cooperation between the polysaccharide matrix and the inorganic component leads to improved encapsulation performance, enhanced stability, or more controlled release behavior.

### 1.3. Methodology

This review followed a structured literature review methodology and used narrative synthesis to integrate the findings. A scoping approach was selected because the evidence base spans heterogeneous polysaccharide chemistries, inorganic phases, fabrication routes, carrier geometries, payload classes, and release-testing conditions, which precludes a single pooled effect estimate but permits structured mapping of material-design relationships. The review question was formulated according to the matrix-filler-processing-payload framework and was as follows: how do (i) polysaccharide identity and functional groups, (ii) inorganic-phase chemistry, morphology, and surface properties, (iii) processing and crosslinking route, and (iv) payload properties determine encapsulation efficiency, retention, structural stability, swelling, barrier behavior, and controlled-release performance?

Two complementary search layers were used. The broad architecture-performance search combined terms for the polysaccharide matrix, inorganic phase, and carrier or release function. A second payload-focused search added terms for volatile compounds, flavors, fragrances, essential oils, cosmetic actives, nutraceuticals, and other bioactive molecules (Table S1). This two-layer strategy was used to retain mechanistic material studies that did not name a specific payload while improving retrieval of application-oriented studies. Search fields were limited to titles, abstracts, and keywords where supported, and no filter based on positive outcomes was applied.

The core search was performed in Scopus, Web of Science Core Collection, and PubMed/MEDLINE for records published from 1 January 2016 to 1 July 2026. Google Scholar was used only as a supplementary discovery tool. The search was updated immediately before manuscript submission. The database searches identified 1486 records in total (698 in Scopus, 521 in Web of Science Core Collection, and 267 in PubMed/MEDLINE), with a further 37 records identified through supplementary Google Scholar searches and backward/forward citation chasing. After removal of 412 duplicate records, 1111 unique records remained for title and abstract screening. Duplicates were removed using DOI, PMID, exact and fuzzy title matching, author, year, and journal data, followed by manual verification. Screening was conducted in two stages: title/abstract screening and full-text eligibility assessment. At this stage, 858 records were excluded as outside the predefined scope. The full texts of 253 articles were assessed against the eligibility criteria presented in Table S2, of which 58 were excluded, primarily because they did not contain a retained inorganic phase, were unrelated to encapsulation or controlled release, lacked sufficient carrier-relevant characterization or did not meet the publication criteria. Ionotropic crosslinking with soluble multivalent ions alone was not classified as an organic-inorganic hybrid unless a discrete inorganic phase remained in the carrier.

Finally, 195 primary studies were included in the core evidence synthesis. Review articles (59) were used separately for background, methodological context, and citation chasing and were not counted as primary included studies.

## 2. Polysaccharides as Organic Polymer Matrices

Recent reviews on polysaccharide nanocarriers and functional polysaccharide systems emphasize that matrix selection should be closely aligned with bioactivity preservation, delivery mechanisms, and the intended food or biomedical application environment. Consistent with this perspective, recent high-impact studies demonstrate, for example, the continued development of alginate- and chitosan-based carrier matrices, showing how cation identity and valence can be exploited to tailor alginate interactions and material properties, while chitosan functionalization can enhance solubility and expand nutraceutical-delivery applications [35,36]. These advances represent important matrix-level developments but should be considered separately from organic-inorganic hybrid systems unless a distinct inorganic phase is retained within the final carrier structure.

### 2.1. Alginate

Alginate is a linear anionic polysaccharide composed of β-D-mannuronic acid and α-L-guluronic acid residues. Its properties depend on molecular weight, the ratio, and sequence distribution of M and G blocks, counterions, concentration, and crosslinking conditions. Calcium alginate networks are formed under mild aqueous conditions and are therefore compatible with enzymes, probiotics, antioxidants, and many volatile or labile active compounds. The same mildness can become a limitation, as ion exchange, high swelling, and limited mechanical strength may accelerate release or destabilize particles in salt-rich, acidic, or surfactant-containing media [1,2,3,4,5,6,9,10].

Recent alginate-focused studies further support the use of alginate as a tunable matrix for encapsulation, controlled release, multifunctional hydrogels, probiotic protection, and nutrition-related delivery systems [13,14,37,38,39,40,41,42,43].

In hybrid systems, alginate carboxylate groups provide coordination sites for cations and interfacial contacts with inorganic phases. Silica, mesoporous silica, halloysite nanotubes, layered double hydroxides, and hydroxyapatite and magnetic particles can be embedded to improve dimensional stability, introduce adsorption sites, increase tortuosity or add additional functions. This is why alginate is a useful reference matrix for comparing other polysaccharides [28,29,30,31,32,33,34,44]. Quantitative data illustrate how hybrid architecture can substantially alter release behavior. In halloysite/alginate hydrogels, salicylic acid loading reached 4.35 wt.%, while 50% of the payload was released after 6–10 h, depending on hydrogel dimensions, compared with approximately 2 h for bare drug-loaded halloysite. The thicker hydrogel additionally exhibited an induction period of about 2 h [44].

Accordingly, alginate performance should not be interpreted as an intrinsic property of the polymer alone, as differences in matrix composition, crosslinking conditions, and release medium can account for substantially different retention and release behavior re-ported across studies [1,2,3,4,5,6,9,10].

### 2.2. Chitosan

Chitosan differs from alginate because its primary amine groups can be protonated, making the polymer cationic under acidic conditions. This enables polyelectrolyte complexes with alginate, pH-responsive multilayers, and chitosan coatings on clay or oxide surfaces [17,45]. Chitosan is also attractive where antimicrobial activity or mucoadhesion is desirable. Its limitations include pH-dependent solubility, batch-to-batch variation in degree of deacetylation, possible sensory effects, and formulation incompatibilities in neutral or alkaline media [45,46].

Recent chitosan- and alginate/chitosan-based studies also show that charge-complementary polysaccharide systems can be tailored for nanoparticle stabilization, mechanical reinforcement, on-demand release, and wound-healing applications [46,47,48,49,50,51,52,53,54]. Chitosan-silica hydrogels and chitosan-coated halloysite systems show how inorganic fillers can compensate for the mechanical and release-control limitations of the polymer phase. Chitosan-alginate systems are particularly useful because the two polysaccharides form complexed shells or coatings that can reduce burst release and introduce pH sensitivity [50,53,54,55]. A quantitative example demonstrates the effect of this architecture on release control. Gentamicin-loaded alginate nanoparticles incorporated into chitosan films had an average diameter of 86 nm, a drug loading of approximately 600 µg/mg, and a loading efficiency close to 100%. Importantly, nanoparticle incorporation reduced the initial gentamicin burst release by 57% at pH 7.4 compared with films containing non-encapsulated gentamicin [52].

Apparently, different outcomes reported for chitosan-containing carriers may arise from differences in degree of deacetylation, polymer ratio, and pH, which alter charge balance and therefore the structure and transport resistance of the resulting carrier [17,45,46,47,48,49,50,51,52,53,54,55].

### 2.3. Cellulose and Nanocellulose

Cellulose and nanocellulose are organic polysaccharide materials that are highly relevant as film-forming matrices and reinforcing phases in multicomponent carriers [56]. Cellulose nanofibrils and cellulose nanocrystals provide high aspect ratio, hydrogen-bonding density, and mechanical reinforcement. When a separate inorganic phase is incorporated, it can additionally improve barrier properties, water resistance, thermal stability, or antimicrobial functionality. These attributes are important for active packaging and for carriers that must function as films or coatings rather than beads. In a cellulose-nanofiber foam containing thyme essential oil, sustained volatile release was achieved, and the TEMPO-oxidized cellulose formulation extended the shelf life of fresh beef by 5 days compared with the untreated control. For cellulose-based systems, performance is particularly dependent on carrier geometry. Therefore, improvements demonstrated in films, coatings, or fibrous networks should not be directly extrapolated to bead-type or highly hydrated carriers [57,58,59,60,61,62,63,64,65,66].

### 2.4. Starch, Maltodextrin, and Other Glucans

Starch, maltodextrin, and related glucans are used in encapsulation, particularly in food and nutraceutical applications, because of their availability, food compatibility, and favorable processing properties [67,68,69,70,71]. Starch can function both as an encapsulating component and as a structural modifier in multicomponent polysaccharide matrices. Starch-filled alginate particles illustrate how the starch fraction can alter the mechanical response of an alginate network [20]. More directly, starch-modified alginate nanoparticles have been investigated as carriers for theophylline and bovine serum albumin, with encapsulation efficiencies of approximately 60–75%, demonstrating the potential of starch-containing matrices for particulate delivery systems [67]. Starch can also participate in organic-inorganic architectures; for example, cationic starch-modified bentonite incorporated into alginate nanocomposites was used to regulate pesticide diffusion, showing how the polysaccharide and layered mineral phases can act cooperatively in controlled-release systems [70].

Maltodextrin is particularly relevant to drying-based encapsulation because of its water solubility and powder-forming ability. In spray-dried roselle extract formulations, combinations of maltodextrin with alginate or carboxymethyl cellulose achieved encapsulation efficiencies of 98.01–98.80%, residual moisture contents of 2.95–3.87%, and powder yields of 50.67–52.80% [21]. These data show that combining maltodextrin with a second polysaccharide can improve the performance of spray-dried carriers. Maltodextrin is also widely employed as a wall material in spray- and freeze-drying of tea, herbal extracts, and other bioactive-rich products, supporting its relevance to scalable food and nutraceutical formulations [68].

The processing route further determines the practical use of these matrices. Spray drying is particularly suitable when a free-flowing dry powder is required, whereas extrusion provides a complementary mild route for producing larger polysaccharide beads or granules [1,21]. Thus, starch- and maltodextrin-based systems can be adapted to different product formats without relying on a single encapsulation technology. Related glucans may also perform more specialized functions: modified dextrin has been used as a pH-responsive coating for mesoporous silica nanoparticles, illustrating how a starch-derived polysaccharide can serve as a functional gatekeeping layer in an organic-inorganic carrier [71]. Overall, these studies reveal a trade-off between processability and release control: although starch- and maltodextrin-based systems are well suited to scalable particulate and powder processing, their moisture sensitivity, rapid hydration, and limited affinity for hydrophobic or volatile compounds may compromise prolonged retention unless the matrix is combined with an additional polysaccharide or an inorganic phase [21,65,67,68,69,70,71].

### 2.5. Pectin, Carrageenan, Xanthan, and Guar

Pectin, carrageenan, xanthan, and guar can be used as primary matrices or as modifiers of alginate- or starch-based systems. Pectin and carrageenan can form ion- or temperature-responsive gels, whereas xanthan and guar mainly control viscosity, suspension stability, and network formation [72]. In hybrids, these polysaccharides are useful when processing requires rheological control, edible status, or compatibility with food and cosmetic formulations [65,73].

Recent work on protein-polysaccharide gels and gum/chitosan coacervates further confirms the value of mixed-polysaccharide systems for essential-oil retention and antioxidant or antimicrobial delivery [74,75,76]. Compared with polysaccharide-only matrices, protein-polysaccharide gels introduce protein-mediated hydrophobic interactions and conformational rearrangements in addition to the hydrogen-bonding and electrostatic interactions characteristic of polysaccharide networks [74]. This more complex interaction pattern can improve interfacial stabilization and modify viscoelastic and moisture-related properties, but it also makes material performance more dependent on the protein-to-polysaccharide ratio and processing conditions. By contrast, polysaccharide-polysaccharide systems are primarily governed by charge complementarity and hydrogen bonding. For example, optimized hydrolyzed karaya gum-chitosan coacervates achieved a complexation yield of 77.30% and an encapsulation efficiency of 65.73% for ginger essential oil [75]. Therefore, pectin-, carrageenan-, xanthan-, and gum-based systems should not be treated as interchangeable matrices, because their contribution to carrier performance depends on whether they act primarily as gel-forming polymers, rheological modifiers, or components of multicomponent complexes [72,73,74,75,76].

A comparative summary of the structural characteristics, functional groups, processing advantages and main limitations of the polysaccharide matrices discussed above is provided in Table 1.

**Figure 2:**
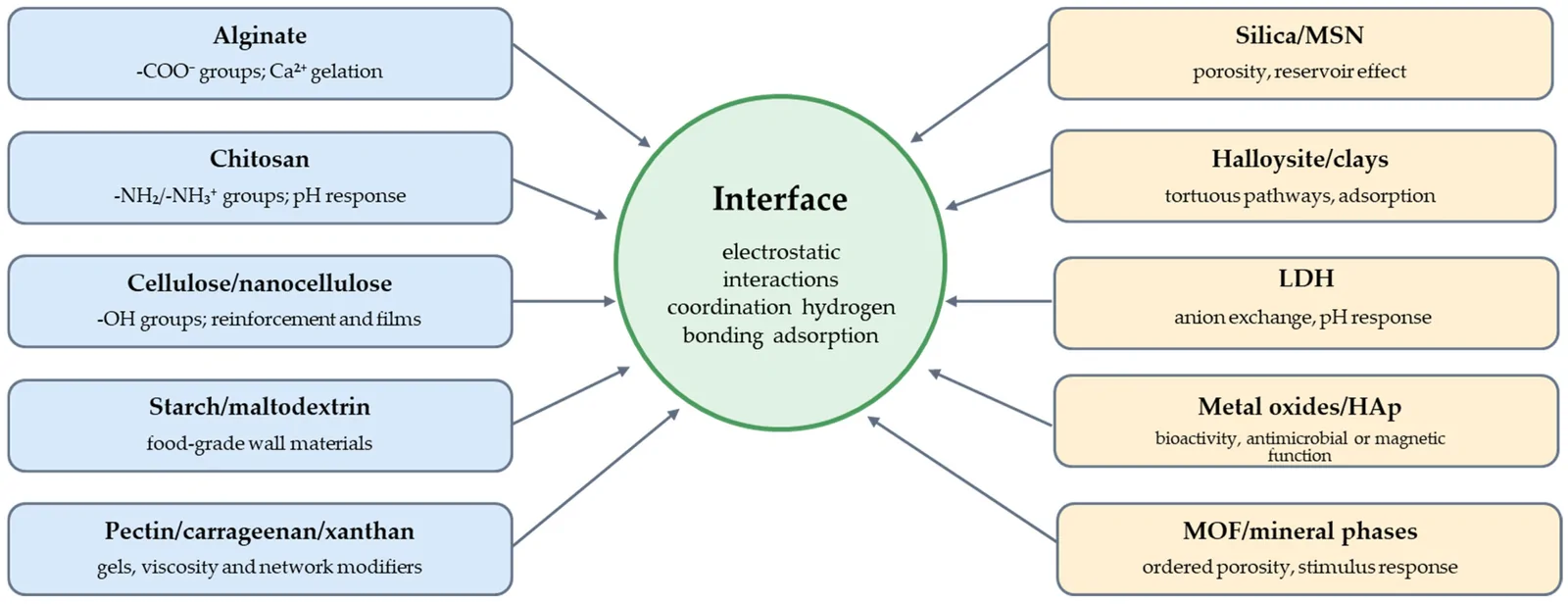
Matrix-filler interactions governing material properties in polysaccharide-based organic-inorganic hybrid carriers. MSN—mesoporous silica nanoparticles; LDH—layered double hydroxides; HAp—hydroxyapatite; MOF—metal-organic framework.

Taken together, the evidence summarized in Section 2 indicates that differences in carrier performance cannot be attributed to polysaccharide identity alone, because matrix composition, carrier geometry, formulation conditions, and payload properties differ substantially among studies. Although the polysaccharides summarized in Table 1 differ in charge, functional groups, gelation behavior, and processing suitability, the final performance of organic-inorganic hybrid carriers depends not only on the polymer matrix itself but also on the nature of its interface with the inorganic phase. Matrix-filler interactions determine filler dispersion, interfacial adhesion, diffusion pathways, swelling behavior, mechanical stability, and the accessibility of adsorption or reservoir sites. The main types of matrix-filler interactions and their contribution to material properties are schematically presented in Figure 2.

The scheme summarizes how selected polysaccharide matrices interact with inorganic components through electrostatic interactions, coordination, hydrogen bonding, and adsorption, thereby affecting porosity, tortuosity, swelling, mechanical stability, encapsulation efficiency, and controlled-release behavior. Therefore, the rational design of polysaccharide-based hybrid carriers requires simultaneous consideration of the organic matrix, inorganic component, and interfacial interactions, as discussed in the following section.

## 3. Inorganic Components in Hybrid Polysaccharide Polymers

Inorganic components play a decisive role in determining the performance of polysaccharide-based organic-inorganic hybrid polymers. While the polysaccharide phase provides biodegradability, processability, water compatibility, and functional groups for gelation, complexation, or film formation, the inorganic phase can introduce porosity, structural reinforcement, adsorption capacity, diffusion barriers, ion-exchange sites, antimicrobial activity, magnetic responsiveness, or bioactivity. Consequently, inorganic fillers should not be considered only as passive additives, as in many hybrid systems, they actively govern swelling behavior, mechanical stability, encapsulation efficiency, encapsulated compound retention, and release kinetics.

The selection of the inorganic component should be matched with the chemical nature of the polysaccharide matrix, the processing route, and the properties of the payload. Silica and mesoporous silica nanoparticles are particularly useful when pore-based loading and reservoir effects are required. Layered clays, halloysite nanotubes, and layered double hydroxides are advantageous when adsorption, interlayer storage, tubular confinement, or diffusion-path elongation is needed [34,86]. Metal oxides and metallic nanoparticles may provide antimicrobial, photocatalytic, or magnetic properties, whereas hydroxyapatite and calcium phosphate phases are mainly selected for bioactive and biomedical materials [87,88]. More advanced mineral phases, including metal-organic frameworks, offer ordered porosity and tunable chemistry, but their integration into hydrated polysaccharide matrices is still developing [89,90]. The functional roles, application relevance, and main limitations of the most important inorganic phases used in polysaccharide-based hybrid carriers are summarized in Table 2. This comparison provides a framework for the following subsections, in which each inorganic component is discussed in relation to its specific contribution to encapsulation, stabilization, and controlled release.

As shown in Table 2, inorganic phases differ not only in chemical composition but also in the way they influence loading capacity, transport pathways, release kinetics, and safety requirements. Overall, the inorganic phase should be selected according to the dominant limitation of the polysaccharide carrier. Silica and mesoporous silica are most useful when pore-based loading and reservoir behavior are required. Clays and halloysite are advantageous when volatile-compound retention and diffusion-path elongation are needed. LDH phases are suitable for ion-exchange and pH-responsive release of charged molecules. Metal oxides introduce antimicrobial or photocatalytic activity, but require strict safety evaluation. Hydroxyapatite and calcium phosphates provide bioactivity and reinforcement, while magnetic particles and MOFs offer advanced responsiveness and high-capacity storage. The most effective hybrid carriers are therefore not those containing the largest amount of inorganic material, but those in which the inorganic phase is rationally matched to the matrix chemistry, processing route, payload properties, and target application. Direct comparison of the available studies indicates that filler selection should be driven by the dominant requirement of the payload rather than by inorganic-phase identity alone. For protein therapeutics, LDH-containing alginate systems are particularly effective when suppression of rapid release is required; in one study, erythropoietin (EPO) release reached 86% after 108 h from alginate alone but only 24% from the corresponding LDH-containing hydrogel, while mechanical performance increased by approximately 5–30-fold [96]. Mesoporous silica provides an alternative reservoir strategy for proteins and other hydrophilic actives, with sustained bovine serum albumin (BSA) delivery reported for up to 24 h [29]. For essential oils and other volatile compounds, tubular or layered fillers are advantageous because they provide additional confinement and diffusion resistance; alginate microbeads containing HNTs, LDHs, or HNT@LDH (halloysite nanotube-layered double hydroxide hybrid) were able to incorporate grapefruit seed oil at loadings of up to 50 wt.% [34]. By contrast, MOF-based systems are more appropriate when high loading and stimulus-responsive release are required; ZIF-8 achieved a payload loading of 14.2 wt.%, followed by sustained release from an alginate composite over 72 h at pH 7.4 [90]. These examples show that the relative advantage of a filler depends on the required balance between payload affinity, release retardation, mechanical reinforcement, and responsiveness.

### 3.1. Silica and Mesoporous Silica Nanoparticles (MSNs)

Silica is among the most frequently used inorganic components in hybrid polysaccharide-based materials because it can be introduced through several routes, including sol-gel chemistry, colloidal silica addition, in situ silicification, incorporation of mesoporous silica nanoparticles, or the use of porous silica fillers obtained from sustainable or low-cost sources. In polysaccharide carriers, silica can strengthen the polymeric matrix, reduce excessive swelling, improve thermal stability and create additional adsorption domains for active molecules [25,28,29,30,31,32,33,55,91,104]. Silica-based systems are also relevant for scalable encapsulation technologies. Polysaccharide/silica hybrid microcapsules prepared by combining ionic gelation with spray drying illustrate how alginate- and chitosan-based matrices can be integrated with in situ silica formation to stabilize capsule structures. This approach connects three elements central to hybrid carrier design: polysaccharide gelation, inorganic structuring, and drying-based processing. It also shows that hybridization should be evaluated together with processability, because the final carrier properties depend on both composition and the fabrication route [28]. Importantly, conventional silica nanoparticles or in situ generated silica nanostructures without deliberately engineered mesoporosity should be distinguished from mesoporous silica nanoparticles (MSNs). In conventional silica-containing hybrids, the inorganic phase mainly contributes through external surface interactions, reinforcement, and modification of matrix swelling or permeability, whereas MSNs additionally provide an internal mesoporous reservoir for active-compound loading [28,29,105].

Recent hybrid systems also show that mesoporous silica can be combined with alginate/chitosan matrices to introduce additional reinforcement, bioactivity, and controlled delivery functions, for example, in bone tissue engineering or detection and targeted drug delivery [92,105,106,107]. The function of mesoporous silica in hybrid carriers is closely related to its pore structure. Ordered mesopores can host active compounds, while the surrounding polysaccharide network controls hydration, particle integrity, and outward diffusion. Consequently, loading in MSNs involves adsorption and confinement within the internal pore network, whereas release additionally requires desorption and diffusion from the mesopores before transport through the surrounding polysaccharide phase [29,107]. This dual-reservoir concept was demonstrated in polysaccharide/mesoporous silica hydrogel beads, where the presence of mesoporous silica improved loading capacity and prolonged release compared with polysaccharide-only systems. Such materials show that the inorganic phase can actively modulate delivery rather than simply reinforce the matrix [29,106].

However, silica integration is not automatically beneficial. Poor dispersion of silica particles can generate defects in films, beads, or capsules, which may accelerate release or reduce mechanical integrity. Silica-rich surfaces may also adsorb the active compound too strongly, limiting its availability during application, or may interact insufficiently with hydrophobic volatile compounds unless the surface is modified. For MSNs, specific surface area determined by BET analysis, pore volume, pore-size distribution, particle size, and zeta potential should be reported together with loading and release data, because these descriptors characterize the available reservoir capacity, pore accessibility, surface properties, and dispersion behavior of the carrier. This distinction is particularly important because high surface area and pore volume can increase loading capacity, whereas pore dimensions and surface charge influence cargo-silica interactions, particle dispersion, and subsequent release [105,106,107].

For hydrophilic compounds, silica may provide hydrogen-bonding and pore-confinement effects. For hydrophobic and volatile compounds, the design is more demanding because efficient retention often requires emulsion templating, surface modification, or the presence of an additional organic domain. Thus, silica-containing polysaccharide hybrids are highly tunable, but their performance depends strongly on the match between pore chemistry, encapsulated-compound properties, and processing conditions [107].

### 3.2. Layered Clays, Halloysite, and Layered Double Hydroxides

Layered clays, halloysite nanotubes, and layered double hydroxides are important inorganic components because they introduce anisotropic morphology and diffusion-controlling structures into polysaccharide matrices. Their main contribution is not simply reinforcement, but also the modification of transport pathways. Plate-like clays can increase the tortuosity of diffusion routes, halloysite nanotubes can act as hollow mineral reservoirs, and layered double hydroxides can provide interlayer spaces with ion-exchange capacity. These features make them especially attractive for systems in which premature leakage, rapid release, or poor retention of the active compound is a major limitation [34,44,93].

Halloysite nanotubes are particularly useful because of their tubular morphology and chemically distinct inner and outer surfaces. The external surface can interact with polysaccharide chains, while the lumen can host active compounds. In hybrid systems, halloysite can therefore serve as a mineral reservoir embedded within a biodegradable polymer matrix. Halloysite-containing polysaccharide systems are especially attractive when release control must combine adsorption, lumen confinement, and a polymeric diffusion barrier [93,94,95,108,109,110].

Layered double hydroxides (LDHs) provide a different mechanism of action. Their positively charged hydroxide layers and exchangeable interlayer anions make them suitable for loading anionic molecules and for pH-sensitive release. When LDH particles are incorporated into alginate beads or films, the resulting hybrids combine ionotropic gelation of the polysaccharide with ion-exchange behavior of the inorganic phase. This makes LDH-containing systems attractive for charged actives and for release environments where pH or ionic composition changes during use [111,112,113]. Moreover, recent examples show the value of comparing different mineral fillers within the same polysaccharide matrix. Alginate microbeads containing grapefruit seed oil were prepared with hybrid fillers based on halloysite nanotubes (HNTs), layered double hydroxides (LDHs), and LDH crystals grown on HNT supports, forming a flower-like HNT@LDH structure [34].

In general, layered and tubular mineral fillers can improve the retention of volatile oils by increasing diffusion-path tortuosity and providing adsorption domains. However, their effectiveness depends strongly on filler loading, dispersion, and compatibility with the polysaccharide network [34,94]. Low filler content may be insufficient to modify release, whereas excessive loading can increase brittleness, viscosity, aggregation, or heterogeneity of the hybrid material. In droplet-based and microcapsule processes, high mineral content may also affect droplet formation and particle size distribution. Therefore, hybrid design should consider filler morphology, aspect ratio, charge, surface chemistry, dispersion stability, and compatibility with the selected processing route [34,44,93,94].

For layered silicates, particle orientation and aspect ratio are additional determinants of barrier performance. High-aspect-ratio platelets can create longer and more tortuous diffusion pathways, particularly when they are well exfoliated and preferentially oriented approximately parallel to the film plane, thereby forcing permeating water, gases, or volatile compounds to diffuse around the impermeable silicate layers. Conversely, random orientation, tactoid formation, or aggregation reduces the effective aspect ratio and continuity of the barrier and can therefore diminish the expected reduction in permeability. In cellulose nanofibril/bentonite films, for example, clay layering and good particle distribution were associated with reduced water and oxygen transport, while the investigated bentonites differed substantially in platelet aspect ratio, illustrating that filler geometry should be considered together with dispersion and interfacial organization [26,113].

Additionally, for food, cosmetic and household applications, clays and halloysite are promising because they can retain natural antimicrobials, essential oils, fragrances, and other volatile compounds. Nevertheless, performance in simple aqueous media does not always translate directly to real formulations. Surfactants, salts, oils, emulsifiers, and complex matrix components can alter adsorption, swelling, and release. For this reason, future studies should evaluate these hybrids not only in buffer solutions but also in representative product environments.

### 3.3. Metal Oxides, Hydroxyapatite, and Magnetic Particles

Metal oxides, hydroxyapatite, and magnetic particles extend hybrid polysaccharide materials beyond conventional encapsulation and release systems. Depending on their chemical identity, particle size, and surface modification, they can introduce antimicrobial activity, photocatalytic behavior, magnetic response, bioactivity, imaging potential, or additional mechanical reinforcement. These functions are attractive in biomedical materials, wound dressings, stimuli-responsive carriers, active packaging, and environmental systems [24,114,115,116].

ZnO, TiO_2_, and Ag-containing systems are among the most widely investigated antimicrobial inorganic phases in polysaccharide matrices. Chitosan is often combined with such particles because it already provides film-forming ability and antimicrobial potential. In food packaging and wound-related materials, inorganic nanoparticles can improve mechanical, barrier, and water-resistance properties, as well as antimicrobial properties, but these benefits must be balanced against potential ion release, particle migration, and oxidative effects on sensitive active compounds [61,63,73,117,118,119].

At the same time, they require more careful safety assessment than many silica- or clay-based fillers, especially when the intended application involves contact with skin, mucosa, biological fluids, or food [120,121]. Furthermore, the incorporation of metal oxides should be interpreted with caution. Particularly, antimicrobial activity may result from ion release, reactive oxygen species generation, direct particle-cell contact, or photocatalytic activation. These mechanisms can be useful in antimicrobial packaging or wound dressings, but they may be problematic for oxidation-sensitive compounds. For example, essential oils, fragrances, and some nutraceuticals can degrade in the presence of reactive oxygen species or under light-activated photocatalytic conditions. Therefore, when metal oxides are used in encapsulation systems, the stability of the encapsulated compound should be evaluated together with antimicrobial or functional performance [122].

Hydroxyapatite and calcium phosphate phases are mainly relevant to biomedical and bioactive materials. In alginate-based systems, mineral phases of this type can improve stiffness, mineral-like character, and osteoconductive potential, while alginate provides mild gelation and shape control. Such systems are less directly related to flavor or fragrance delivery, but they illustrate how an inorganic bioactive phase can simultaneously influence material morphology, mechanical response, and release behavior [88,98,100,123,124].

Magnetic particles, especially Fe_3_O_4_-based nanoparticles, introduce another design function. They allow magnetic separation, externally guided positioning, magnetically assisted release, or local heating under alternating magnetic fields. Magnetic alginate or chitosan-based hydrogels are increasingly studied for targeted delivery, imaging, tissue engineering, and environmental applications. In controlled-release systems, magnetic particles may be useful when remote triggering, recovery of the carrier, or spatial control is required [125,126,127,128]. However, their use in food, cosmetic, and household products is more restricted and requires detailed evaluation of migration, exposure route, particle stability, and regulatory acceptability. The presence of a biocompatible polysaccharide matrix does not eliminate the need to assess the inorganic nanophase. The final safety profile depends on particle chemistry, size, dissolution, aggregation, migration, degradation products, and application route. For this reason, studies involving metal oxides, hydroxyapatite, or magnetic particles should report not only release performance but also particle immobilization, inorganic species release, and relevant toxicity or compatibility data.

### 3.4. Metal-Organic Frameworks and Advanced Mineral Phases

Metal-organic frameworks are an emerging class of porous crystalline materials with high surface area, ordered pore structures, and tunable chemical functionality. Their integration with polysaccharide matrices is attractive because the polymer phase can improve processability, reduce powder-handling problems, provide biocompatibility and control the macroscopic form of the material. The MOF phase, in turn, can act as a high-capacity reservoir for active molecules and may introduce pH-responsive release, selective adsorption, or antibacterial activity. However, compared with silica, clays, or hydroxyapatite, MOF-containing polysaccharide carriers are still at an earlier stage of development.

Recent reviews and experimental studies on MOF-based hydrogel materials emphasize that MOFs alone may suffer from limited processability, particle-handling difficulties, and insufficient stability in aqueous or biological environments. Embedding MOFs in hydrogels can improve shapeability, mechanical integrity, and application convenience while retaining part of the porosity and functionality of the MOF phase. This concept is relevant for polysaccharide-based systems because alginate and related polymers can form hydrated networks capable of immobilizing MOF particles and regulating swelling-controlled release [89,90,102,129].

MOF-polysaccharide composites, including ZIF-8/alginate systems, illustrate the potential of this approach for drug-carrier design. In such materials, the polysaccharide phase improves handling, shaping, and hydration control, while the MOF phase provides ordered porosity and adsorption sites. Although these studies are mainly biomedical or proof-of-concept, they demonstrate design principles that may be transferred to other bioactive compounds if water stability, metal ion release, linker safety, and regulatory constraints are addressed [90,112,130,131,132].

For alginate and other polysaccharides, MOF integration may provide high loading, ordered porosity, and responsive release, but water stability, metal ion release, organic linker safety, and compatibility with food or cosmetic uses remain major constraints [90]. At present, MOF-based polysaccharide hybrids are most convincing in biomedical, environmental, and proof-of-concept controlled-release studies. Their broader use in food, fragrance, or cosmetic formulations will require MOFs with benign composition, aqueous stability, low migration risk, scalable synthesis, and compatibility with regulatory expectations.

## 4. Fabrication Strategies

Fabrication strategy is a key factor determining whether a given polysaccharide-inorganic composition forms a hydrogel bead, microcapsule, dry powder, film, coating, aerogel, or fibrous mat. Consequently, structure-property relationships in hybrid polysaccharide carriers cannot be interpreted solely on the basis of chemical composition. The same alginate-silica, chitosan-clay, or starch-mineral formulation may exhibit different swelling behavior, mechanical stability, encapsulation efficiency, and release kinetics, depending on whether it is processed by ionotropic gelation, spray drying, extrusion, casting, electrospinning, or sol-gel/in situ mineralization.

Therefore, fabrication strategy should be selected by balancing payload compatibility, material composition, target geometry, scalability, and cost. Ionotropic gelation and polyelectrolyte complexation are particularly suitable for alginate-, pectin-, and carrageenan-based systems containing silica, clays, LDH, or hydroxyapatite and for hydrophilic or labile payloads, including proteins and probiotics. These predominantly aqueous, low-temperature processes require relatively low capital investment, although their throughput is generally low to moderate unless continuous nozzle- or extrusion-based processing is applied [2,3,97]. Sol-gel and in situ mineralization are more appropriate for alginate- or chitosan-based hybrids containing silica or hydroxyapatite and for payloads tolerant of precursor chemistry. However, precursor control, pH adjustment, aging, and washing increase process complexity and restrict scalability [32]. Spray drying is particularly advantageous for starch/maltodextrin-, alginate/pectin-, or chitosan-based systems containing silica or clays and intended for flavors, fragrances, and nutraceuticals. It provides high-throughput continuous production and relatively low unit costs at an industrial scale, although heat and air exposure may compromise volatile or oxidation-sensitive compounds [28]. Extrusion and continuous gelation are also readily scalable and comparatively inexpensive, especially for alginate-, pectin-, or carrageenan-based granules used in food, probiotic, or household applications, but generally produce larger particles with less precise size control. Casting and coating are most appropriate for cellulose/nanocellulose, chitosan, alginate, or starch matrices containing clays or metal oxides when continuous films, packaging layers, or textile coatings are required. Laboratory casting is low-throughput, whereas industrial coating processes can achieve medium-to-high production scales at low-to-moderate cost. Electrospinning is better suited to alginate blends, chitosan, or cellulose derivatives containing nanoclays, silica, or metal oxides for fibrous wound dressings and patch-type systems, but typically involves lower throughput and higher capital and energy requirements [133,134,135]. Overall, spray drying and extrusion offer the most favorable combination of scalability and cost, whereas sol-gel processing and electrospinning are more appropriate when their specific structural or functional advantages justify greater process complexity.

**Figure 3:**
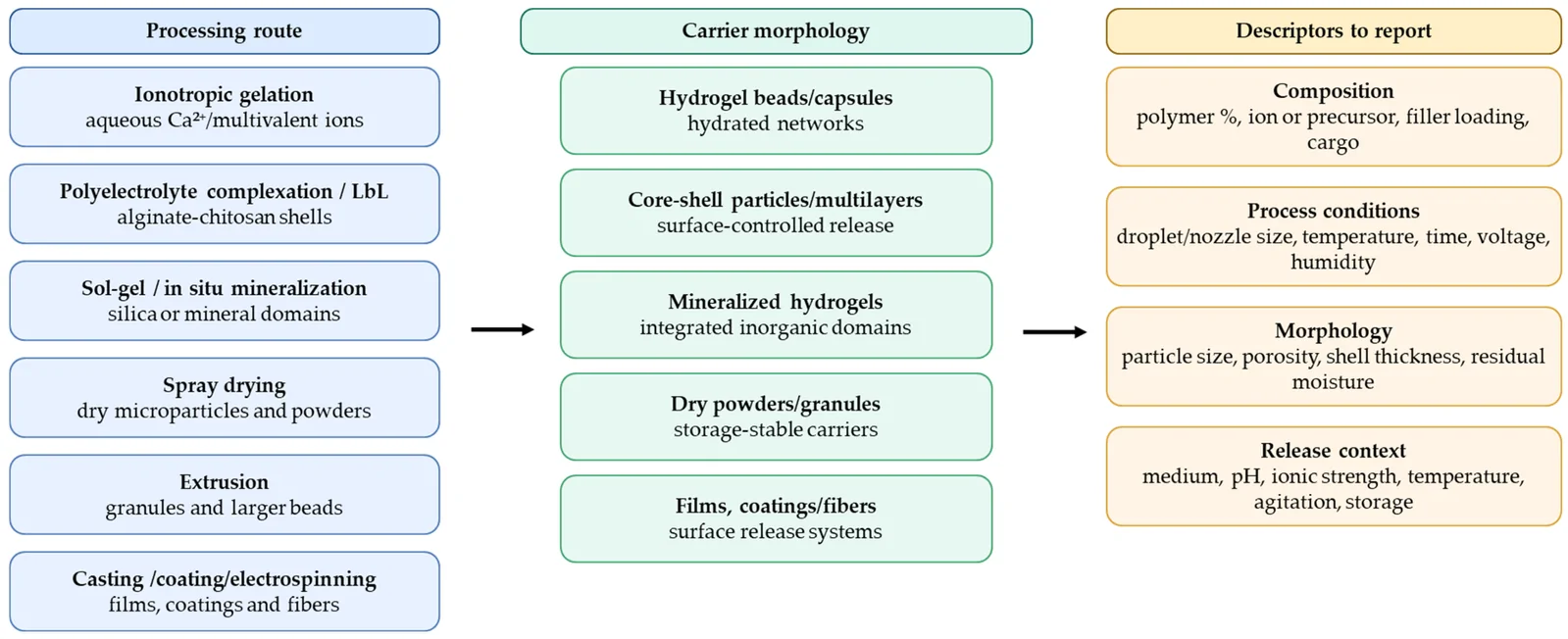
Processing routes and descriptors for reproducible polysaccharide-based organic-inorganic hybrid carriers.

Nevertheless, cross-method comparisons should be interpreted cautiously because individual studies employ different matrices and payloads and report different performance endpoints, including encapsulation or loading efficiency, viability retention, and barrier properties. Numerical values are therefore not directly comparable across fabrication strategies. More relevant than identifying the method with the highest isolated efficiency is determining whether a given process meets the application-specific performance requirements while remaining scalable and compatible with the active compound. Figure 3 maps the principal processing routes to the resulting carrier morphologies and the minimum process descriptors required for reproducible comparison.

Processing controls and carrier morphology should be reported together with encapsulation and release conditions. Therefore, Table 3 intended as a decision framework provides a practical overview of fabrication strategies, typical products, main advantages, critical descriptors, and application relevance.

Table 3 should therefore be interpreted as a decision matrix rather than a ranking of fabrication methods, because no fabrication route is universally superior. The final choice should be based on the combined constraints of matrix and filler processability, payload sensitivity, target geometry, production scale, and required release mode.

### 4.1. Ionotropic Gelation and Polyelectrolyte Complexation

Ionotropic gelation is most useful when mild aqueous processing is the primary requirement, particularly for alginate and other ionically gel-forming polysaccharides. From a fabrication perspective, the relevant distinction is between external gelation, where crosslinking proceeds from the droplet surface inward, and internal gelation, where ions are generated within the polymer phase and can produce a more homogeneous network. Both routes are simple and low-cost, but crosslinking gradients and droplet-generation conditions can affect particle-size uniformity and encapsulation performance [1,2,3,10,147].

For hybrid carriers, inorganic particles can be dispersed in the polymer feed before gelation, enabling direct entrapment of silica, halloysite, LDH, hydroxyapatite, magnetic particles, and related phases. The main fabrication constraint is maintaining a stable, processable suspension: sedimentation, aggregation, or excessive viscosity can produce heterogeneous filler loading and inconsistent beads. Accordingly, feed rheology, filler concentration and dispersion, droplet size, crosslinker concentration, and residence time should be controlled [98,148].

Polyelectrolyte complexation, most commonly between anionic alginate and cationic chitosan, provides core-shell or multilayer structures without introducing a separate high-temperature step. At the molecular level, shell formation begins when protonated amino groups of chitosan bind to negatively charged carboxylate groups of alginate through electrostatic attraction [17,54]. Because chitosan chains must diffuse from the bulk solution to the alginate interface before complexation occurs, deposition may become diffusion-controlled. Progressive occupation of the available anionic sites leads first to charge neutralization and, at higher surface coverage, to charge reversal, which enables subsequent deposition of oppositely charged layers in multilayer assemblies [45]. Its principal fabrication advantage is the ability to modify surface charge and create a diffusion-controlling shell after bead formation. Performance is highly process-dependent: pH, charge ratio, order of addition, mixing regime, and coating thickness affect particle size and release behavior [17,45,47,49,52,53,54].

Beyond food, cosmetic, and biomedical applications, alginate/chitosan matrices are also being explored as controlled-release platforms for agricultural biostimulants [149]. However, this example is included here as contextual evidence for mixed-polysaccharide controlled-release systems rather than as a genuine organic-inorganic hybrid. Similarly, the polysaccharide-only systems, which can also be combined with fibrous substrates, are included in this review as mechanistic and processing context, as the same polymer pair can give different release profiles depending on the formation pathway [49,52,53,54].

Moreover, recent optimization studies indicate that alginate-chitosan spheres can also be designed using statistical and machine-learning approaches to improve essential-oil encapsulation and release performance. A representative example is the study by Taouzinet et al., in which response surface methodology (RSM) and an artificial neural network (ANN) were compared for optimizing alginate-chitosan particles containing *Rosmarinus officinalis* essential oil. The ANN achieved an R^2^ of 0.999, compared with 0.991 for RSM, and the optimized formulation contained 2 g of alginate, 1.05 g of chitosan, and 80 μL of essential oil [150]. The resulting particles were subsequently assessed in terms of entrapment efficiency, swelling behavior, component interactions, and lipid peroxidation, linking computational optimization with physicochemical and functional performance. This example demonstrates the potential of data-driven formulation design, although broader validation across different polysaccharide matrices, payloads, and processing conditions is still required before such models can be generalized.

Despite many advantages, ionotropic gelation and polyelectrolyte complexation have important limitations. Droplet-based gelation may lead to broad particle-size distribution unless assisted by microfluidics, vibrating nozzles, or controlled extrusion. Polyelectrolyte shells may be sensitive to pH, ionic strength, and competing ions. In addition, high loading of hydrophobic or volatile compounds often requires preliminary emulsification, because the aqueous alginate phase alone has limited affinity for nonpolar active compounds. For this reason, gelation-based systems for flavors, fragrances, and essential oils should include information on emulsion stability, oil-droplet size, surfactant type, and possible interactions with the inorganic phase.

### 4.2. Sol-Gel and In Situ Mineralization

Sol-gel chemistry and in situ mineralization are used when the aim is to generate inorganic domains inside, around, or in close contact with a polysaccharide matrix. In silica-containing systems, hydrolysis and condensation of alkoxysilane precursors can create mineral networks within hydrogels, beads, films, or capsules. Compared with simple addition of preformed particles, in situ formation may improve interfacial contact between the polymer and inorganic phase and may produce a more continuous mineral distribution. This can be beneficial for mechanical stability, swelling control, and retention of active compounds [30,32].

The main advantage of sol-gel processing is the possibility of tuning the inorganic phase during material formation. Precursor concentration, water-to-precursor ratio, pH, catalyst type, aging time, and drying conditions influence silica condensation, pore formation, and interfacial interactions. In polysaccharide matrices, hydroxyl, carboxyl, and amino groups can participate in hydrogen bonding or electrostatic interactions with developing inorganic domains. Such interactions may improve immobilization and reduce phase separation. However, the same chemistry can also create constraints when the introduced active compound is sensitive to pH, alcohol formation, hydrolysis products, or prolonged washing [151,152].

Among the representative silica sol-gel systems discussed here, acid-catalyzed processing is explicitly used in the TEOS-based silica/sodium alginate system [32], although the cited hybrid fabrication routes are not all directly comparable conventional acid- or base-catalyzed sol-gel syntheses. Catalyst selection is nevertheless an important design parameter because acidic and basic conditions alter the relative rates of silane hydrolysis and condensation. Consequently, it affects the degree of silica network formation, pore structure, particle aggregation, and polymer-silica interfacial organization.

For food-grade or food-contact formulations, the suitability of sol-gel processing should therefore be evaluated at the level of the complete synthesis route rather than inferred from the food compatibility of the polysaccharide matrix alone. Alkoxysilane-based routes may involve precursors such as tetraethyl orthosilicate (TEOS), alcohol-containing reaction media, and acid or base catalysts. For example, the silica/sodium alginate hybrids described by D’Angelo et al. were synthesized using TEOS in a water/methanol medium under acid-catalyzed conditions [32]. TEOS was also used as the silica precursor in the polysaccharide/silica microcapsules reported by Elzayat et al. [28]. Consequently, the toxicological acceptability of the precursor system and the possible presence of unreacted precursor, residual solvent, catalyst, and other low-molecular-weight process-derived species should be considered when such approaches are transferred to food-related applications. Washing and drying can reduce residual processing chemicals, but these operations increase process complexity and may simultaneously promote loss of sensitive or volatile active compounds. Therefore, residual-process chemicals should be analytically controlled rather than assumed to be completely removed.

Polysaccharide/silica microcapsules prepared by combining alginate/chitosan gelation, silica formation, and spray drying provide a relevant example of an integrated fabrication route. This approach links mild ionic gelation with a scalable drying step and produces water-resistant particles [28]. From the perspective of this review, it demonstrates that fabrication strategies are rarely isolated: ionic gelation, in situ silica formation, and spray drying can be combined to create carriers with improved structural stability.

For volatile compounds like flavors and fragrances, sol-gel and mineralization routes require special caution. Volatile molecules can be lost during precursor mixing, gelation, aging, washing, or drying step. In addition, some fragrance or flavor compounds may interact with silanol groups or mineral surfaces, causing either excessive retention or altered release. Therefore, when sol-gel approaches are used for volatiles loading, it is important to quantify not only encapsulation efficiency immediately after preparation but also retention after processing and storage [32]. Headspace analysis, thermogravimetric measurements, and release tests under realistic humidity or temperature conditions are especially useful for such systems.

In situ mineralization can also be extended beyond silica. Hydroxyapatite, calcium phosphate, carbonate, or mixed mineral phases can be generated within polysaccharide networks to produce bioactive or reinforced carriers [88,153]. These systems are relevant when the carrier must combine encapsulation with pH buffering, bioactivity, mineral-like reinforcement, or additional adsorption sites. In situ mineralization can generate strong interfacial contact between the organic and inorganic phases, but it may also expose sensitive active compounds to changes in pH, ionic strength, or reactive precursors. These routes are promising for biomedical applications, but less universal for food, cosmetic, and fragrance systems because mineralization often requires pH changes, ionic precursors, or aging conditions that may not be compatible with sensitive active compounds. The choice of mineralization strategy should be guided by the stability of the active compound and by the intended release environment, specifically when the active compound is volatile, oxidation-sensitive, or structurally labile.

An additional distinction is required between biomedical biocompatibility and food-grade acceptability. Favorable cytocompatibility or bioactivity of a mineralized polysaccharide carrier does not by itself establish its suitability for ingestion or food contact, because the relevant exposure routes and safety criteria are different. For food-contact systems, migration of the inorganic phase or released ions into the food should be evaluated under conditions representative of the intended product and storage conditions. For example, in a soluble soybean polysaccharide/TiO_2_ bionanocomposite film developed for food application, TiO_2_ was not detected in bread stored in contact with the film for six months [118]. This result demonstrates that effective immobilization and very low migration can be achieved in a specific polysaccharide-inorganic system, but it should not be generalized to other fillers, particle sizes, loadings, foods, or processing routes. Accordingly, food-oriented sol-gel and mineralized carriers should be assessed with respect to the grade and regulatory acceptability of both the polysaccharide and inorganic components, residual precursors and solvents, migration or leaching of inorganic species, and consumer exposure under realistic conditions [58,87,118]. These considerations are particularly important for directly ingested carriers, for which the inorganic phase and residual processing chemicals become part of the exposure scenario, whereas in food-contact packaging the principal concern is their potential transfer from the carrier into the food.

### 4.3. Spray Drying, Extrusion, and Powder Technologies

Spray drying and extrusion are the routes in this section with the clearest scale-up potential. Both can operate continuously or semi-continuously, but their stress profiles differ: spray drying combines atomization, hot gas, and rapid solvent removal, whereas extrusion is dominated by shear, nozzle geometry, and subsequent gelation or drying. The distinction is important for volatile, oxidation-sensitive, and structurally labile payloads.

Spray drying converts emulsions, suspensions, or solutions into dry powders within short residence times and is attractive for flavors, fragrances, and nutraceuticals because of its high throughput and relatively low processing cost. Its main limitation is the simultaneous exposure of the active compound to heat and air; retention therefore depends on feed stability, wall-material composition, inlet/outlet temperature, atomization, residual moisture, and surface-associated cargo. For hybrid powders, silica, clays, or halloysite can improve moisture resistance or retard release, but poor filler dispersion or strong cargo adsorption can offset these benefits. Volatile systems therefore require assessment of sur-face oil and headspace retention in addition to total encapsulation efficiency [28,141,154,155,156,157].

Extrusion is simpler and less thermally demanding than spray drying and is particularly suitable for alginate beads, food granulates, and household carriers. It can be implemented continuously with relatively low equipment complexity, but nozzle-based formation usually gives larger particles and less precise size control than atomization or microfluidics. Filler sedimentation and axial variation in active-compound distribution are additional scale-up concerns [1,144].

Powder technologies may also include freeze drying, spray freeze drying, fluidized-bed coating, and agglomeration. Freeze drying can preserve temperature-sensitive compounds, but it is slower, more expensive and may produce highly porous structures with rapid rehydration and release. Fluidized-bed coating can add protective layers to preformed particles, allowing better control of release and moisture sensitivity. These approaches are particularly useful when the carrier must remain stable as a dry product during storage but release the active compound after hydration, mechanical stress, or exposure to humidity [158,159].

### 4.4. Casting, Coating, and Electrospinning

Casting and coating are central fabrication strategies when the target product is a film, patch, active coating, textile finish, or packaging layer. Unlike beads and powders, films and coatings are designed to function as continuous barriers or interfacial layers. Their performance depends strongly on thickness, drying conditions, humidity, plasticizer content, filler dispersion, and adhesion to the substrate. In polysaccharide-based hybrids, inorganic particles can improve mechanical strength, oxygen barrier properties, thermal stability, or antimicrobial function, but excessive filler loading may increase brittleness or reduce transparency [58,160].

Casting is widely used at a laboratory scale because it is simple and allows direct comparison of formulations. A polysaccharide solution containing a plasticizer, an active compound, and an inorganic filler is poured onto a surface and dried under controlled conditions. This method is useful for screening polymer-filler compatibility and barrier properties, but it does not always translate directly to industrial coating or film-forming processes. Drying rate, film thickness, and humidity strongly influence polymer chain organization, filler migration, and final morphology. Therefore, casting studies should report not only composition but also wet-film thickness, drying temperature, relative humidity, and conditioning protocol before testing [59,64,146].

Coating methods, including dip coating, spray coating, and LbL deposition, are useful when the hybrid material must be deposited on an existing substrate. LbL assembly is particularly attractive for alginate-chitosan systems because it allows alternating deposition of oppositely charged polysaccharides and creates films with controlled surface charge and pH response [161]. Chitosan/alginate multilayers on electrospun PLGA nanofibers demonstrate how a mechanically stable scaffold can be combined with a responsive polysaccharide coating to control release [45].

Electrospinning creates fibrous mats with a high surface area, interconnected porosity and tunable fiber morphology. These features are attractive for wound dressings, transdermal systems, active packaging, filtration materials, and controlled release patches. Recent reports on alginate-based electrospun nanofibers emphasized that electrospun alginate systems can be designed for pulsatile, sustained, biphasic, stimulus-responsive, and targeted release. However, pure alginate has limited spinnability because of its chain rigidity, ionic character, and strong hydrogen-bonding interactions. For this reason, alginate is often blended with synthetic or semi-synthetic polymers such as PEO, PVA, or PLGA, or modified chemically to improve processability [134,145]. These mixed systems illustrate a design trade-off relevant to encapsulation. Synthetic components such as PLGA, PEO, or PVA can improve processability or provide mechanically stable support, whereas polysaccharide layers can introduce responsive release behavior [45,134,145]. Because purely synthetic polymer carriers were excluded from the predefined scope of this review (Table S2), a universal quantitative comparison of encapsulation efficiency with fully synthetic systems is not attempted. Electrospinning can also be combined with inorganic fillers. Nanoclays, halloysite nanotubes, silica, metal oxides, or magnetic particles may be incorporated into fibers to improve mechanical performance, introduce antimicrobial activity or act as reservoirs for active compounds. However, filler addition can alter conductivity, viscosity, and surface tension of the spinning solution, which may change fiber diameter, bead formation, and mat uniformity. Therefore, electrospun hybrid systems require detailed reporting of polymer concentration, solvent system, voltage, flow rate, collector distance, humidity, filler dispersion, and post-treatment [162].

For volatile compounds, casting, coating, and electrospinning present different challenges than bead or powder technologies. A high surface area can accelerate evaporation, while long drying times may reduce retention. At the same time, film and fiber architectures may be advantageous when controlled release from a surface is required, such as in active packaging, textile fragrance systems, cosmetic patches, or antimicrobial coatings. In these applications, release should be tested under conditions that mimic actual use, including humidity cycling, mechanical deformation, contact with oils or surfactants, and exposure to air [46,163,164]. Recent studies on essential oil film underline that retention in polysaccharide matrices depends on emulsion stability, film formation, oil-polymer affinity, and storage conditions [165,166,167,168,169].

Overall, fabrication strategy must be selected according to the target carrier format and application environment. Ionotropic gelation is suitable for mild aqueous encapsulation, polyelectrolyte complexation for responsive shells, sol-gel routes for integrated mineral networks, spray drying for scalable powders, extrusion for larger granules, and casting or electrospinning for films and fibrous systems. No single processing method is universally superior. The most effective strategy is the one that matches matrix chemistry, inorganic phase, payload sensitivity, desired morphology, and release trigger.

## 5. Structure-Property Relationships

The performance of polysaccharide-inorganic hybrid carriers emerges from interactions occurring across several structural levels. At the molecular scale, functional groups of the polysaccharide matrix determine ionic crosslinking, hydrogen bonding, coordination with metal ions, and electrostatic complexation. At the interfacial level, the compatibility between the polymer matrix and the inorganic phase controls adhesion, filler immobilization, and the formation of continuous or defective structures. At the colloidal scale, the dispersion, size, morphology, and surface chemistry of the inorganic component determine whether the filler reinforces the matrix, creates adsorption domains or acts as a defect-generating aggregate. At the macroscale, carrier geometry controls water uptake, mechanical integrity, storage stability, and release directionality. This multiscale character explains why chemical composition alone is insufficient for comparing encapsulation systems [25,28,46,55,160,170,171,172].

In polysaccharide-based hybrid polymers, structure-property relationships are therefore governed by the cooperation between matrix chemistry, filler architecture, and processing-induced morphology. A calcium alginate bead, a chitosan-coated alginate capsule, a nanocellulose/clay film, and a spray-dried starch-silica powder may contain similar functional groups or inorganic components, but their performance will differ because their network density, porosity, water content, surface area and diffusion geometry are different [28,172]. The same inorganic filler can improve mechanical stability in one system, act as a reservoir in another or create structural discontinuities if it is poorly dispersed [173,174,175]. For this reason, structure-property analysis should integrate molecular interactions, microstructure, carrier format, and release environment rather than treating these parameters separately.

At the matrix-filler interface, electrostatic interactions, coordination, hydrogen bonding, and adsorption can improve filler immobilization, modify swelling behavior, strengthen the matrix and regulate access of water or active molecules to internal reservoirs. These interactions are essential for stabilizing hybrid carriers, but they must be balanced carefully. If the matrix-filler interaction is too weak, the inorganic phase may aggregate, migrate or generate defects. If the interaction between the active compound and the inorganic phase is too strong, release may become incomplete or too slow for the intended application [44,176]. Thus, the optimal hybrid carrier is not necessarily the system with the strongest interaction, but the one in which interfacial adhesion, water transport, and active-compound affinity are matched to the desired release profile. Nevertheless, direct numerical comparison of interfacial interaction strength across the reviewed systems is difficult because the available studies do not use a standardized interaction-energy parameter. In alginate-based systems, carboxylate groups provide sites for coordination and electrostatic interactions with inorganic phases, whereas in chitosan-based systems protonated amino groups predominantly support electrostatic association [12,17,45]. Therefore, interfacial strength should be interpreted using system-specific physicochemical descriptors rather than as a universal numerical value.

A practical comparison of the main structure-property descriptors relevant to polysaccharide-based organic-inorganic hybrid carriers is provided in Table 4. These descriptors are particularly important when release data from different studies are compared, because variations in particle size, swelling ratio, filler dispersion, humidity response, or payload localization can explain differences that would otherwise be incorrectly attributed only to chemical composition.

Mechanical reinforcement is one of the most frequently cited benefits of adding inorganic phases to polysaccharide matrices, but it depends strongly on filler dispersion and interfacial adhesion. Well-dispersed silica, clay platelets, or mineral particles can increase stiffness, reduce deformation and improve dimensional stability. By contrast, poorly dispersed fillers can act as stress concentrators and generate weak points that accelerate cracking, swelling, erosion, or leakage of the encapsulated compound. The difference between reinforcement and defect formation is especially important for hydrogel beads, microcapsules, and films, where local structural failure can lead to uncontrolled release [186]. The mechanical behavior of alginate-based materials also depends on crosslinking architecture, not only on filler addition. Calcium alginate networks are widely used because they form under mild aqueous conditions. However, their mechanical stability may be limited by high water content and ion exchange. More complex crosslinking strategies can substantially alter network density, deformation behavior, and swelling. For example, reconstructed alginate hydrogels have shown that processing-induced densification followed by ionic crosslinking can lead to strong and stiff networks [187]. Similarly, Fe^3+^-cross-linked alginate hydrogels illustrate how the coordination mode of trivalent ions can affect mechanical strength, porosity, swelling, and physicochemical stability [188]. These examples show that mechanical reinforcement in polysaccharide-based hybrids is controlled by the combined effects of crosslinking, water content, filler dispersion, and network organization.

Barrier properties are also strongly dependent on material architecture. In dry films clays and silica can reduce oxygen or vapor transport by increasing path tortuosity and creating dense hydrogen-bonded or mineral-reinforced networks [189,190]. This effect is particularly relevant for active packaging, cosmetic films, and surface-release systems, where the carrier must slow oxygen, moisture, or volatile loss during storage. Cellulose- and nanocellulose-based hybrid films are useful in this context because they combine film-forming ability, mechanical support, and opportunities for inorganic reinforcement [191].

However, barrier performance is highly sensitive to humidity. Many polysaccharide films perform well under dry or moderately humid conditions, but water plasticization can reduce intermolecular interactions, increase chain mobility and open diffusion pathways. This is particularly important for hydroxyl-rich networks such as nanocellulose, starch, and maltodextrin-based films [192]. Under high relative humidity, a film that acts as an oxygen barrier in dry conditions may become more permeable because absorbed water disrupts hydrogen bonding and increases free volume. For volatile compounds, this effect is critical because storage loss may occur before the intended release step [157]. Therefore, barrier properties should be reported under humidity conditions that reflect the real application environment rather than only under standard dry testing conditions.

Swelling and porosity are double-edged features in controlled-release systems. A highly porous bead, aerogel, or hydrogel can incorporate more active compound and allow rapid hydration, but it can also accelerate release by increasing water access and shortening diffusion pathways. Conversely, a dense shell or compact film can reduce initial burst release, but may limit loading or prevent complete release [180,193]. The optimal porosity therefore depends on whether the goal is rapid activation, sustained release, delayed release, or long-term storage retention. For hydrogel beads, pore size and swelling ratio are particularly important because they determine both water influx and active compound diffusion [194]. For films and coatings, microvoids and cracks may be more relevant than total porosity because they can act as preferential channels for vapor transport [195].

Inorganic reservoirs can partly decouple loading capacity from release rate. Mesoporous silica, halloysite nanotubes, and layered double hydroxides can host active compounds within pores, lumens, or interlayer spaces, while the surrounding polysaccharide phase controls hydration and diffusion. This architecture is especially useful, as a highly hydrated polysaccharide gel would otherwise release the active compound too quickly. Alginate beads containing mesoporous glass and curcumin illustrate this concept, because the mesoporous inorganic phase can stabilize and deliver the active compound while alginate provides a hydrated macroscopic carrier [196]. Similar principles are relevant for essential oils and fragrances, where mineral pores or tubular reservoirs can reduce evaporation and improve retention [34,197,198].

For hydrophobic flavors, fragrances, and essential oils, the most effective architecture is often not the most hydrophilic gel. Hydrophilic networks absorb water readily, but they may have limited affinity for nonpolar volatile compounds. As a result, hydrophobic active compounds may migrate to interfaces, separate during processing or evaporate during drying and storage. Hybrid architectures can address this limitation by combining three functions: adsorption in an inorganic reservoir, diffusion control by the polysaccharide matrix, and moisture-dependent release [25]. Essential-oil encapsulation studies consistently show that carrier type, particle structure, release medium, and environmental conditions strongly affect release behavior [63,199,200,201,202,203].

The relationship between swelling and release is also affected by environmental triggers. pH can change the ionization state of alginate, chitosan, and pectin; ionic strength can weaken calcium alginate through ion exchange; surfactants can solubilize hydrophobic active compounds and alter partitioning; and temperature can modify diffusion, viscosity, and vapor pressure. These factors are often studied separately, but in real formulations they act simultaneously. Cosmetic emulsions, detergent matrices, food products, and active packaging environments contain salts, surfactants, oils, proteins, plasticizers, or humectants that may change both matrix swelling and active compound mobility. Therefore, structure-property analysis should include formulation-relevant media whenever possible [183,184,204,205].

The most informative structure-property studies are those that connect material descriptors with performance outcomes. Filler loading should be linked with dispersion and mechanical behavior, not only with release rate. Swelling ratio should be reported together with pore morphology and crosslinking conditions. Barrier properties should be interpreted together with humidity response. To improve cross-study comparability, these relationships should be expressed quantitatively where possible, using metrics such as mechanical strength or modulus, swelling ratio, permeability or barrier coefficients, initial burst fraction, characteristic release time (e.g., t50 when reported), headspace retention, and filler or ion leaching.

Matrix-filler-payload interactions directly govern controlled-release behavior, because strong adsorption within mesopores or clay interlayers can improve payload retention while also hindering subsequent release. For volatile or oxidation-sensitive compounds, this balance should therefore be evaluated through storage retention, payload localization, and application-relevant release rather than encapsulation efficiency alone [157,176]. Release profiles should be discussed in relation to particle size, shell thickness, porosity, and active compound-matrix affinity. Such integrated analysis is necessary because the same release curve can result from different mechanisms, including diffusion through a hydrated matrix, erosion, desorption from inorganic pores, ion exchange, swelling-controlled transport, or partitioning into the surrounding medium.

Overall, polysaccharide-inorganic hybrid carriers should be evaluated as multiscale materials rather than as simple mixtures of a biopolymer and a filler. Their final properties are determined by matrix chemistry, inorganic phase morphology, interfacial interactions, processing history, product geometry, and environmental response. For volatile and bioactive compounds, the most promising systems are those that combine sufficient payload affinity during storage with controlled release under use conditions. This balance requires rational design of the matrix-filler interface, careful control of water uptake, and systematic reporting of structural descriptors.

## 6. Encapsulation Efficiency, Retention, and Controlled Release

Encapsulation efficiency is one of the most frequently reported parameters in studies on polysaccharide-based hybrid carriers, but it should not be interpreted as a standalone indicator of material performance. A system may show high initial encapsulation efficiency immediately after preparation, yet exhibit poor retention during drying, storage, or formulation processing. Conversely, a carrier may retain the active compound very efficiently, but release it incompletely or too slowly under use conditions. This distinction is particularly important for volatile, oxidation-sensitive, or hydrophobic compounds, including flavors, fragrances, essential oils, and selected cosmetic or nutraceutical actives. For such payloads, the practical value of a carrier depends not only on how much compound is initially incorporated but also on how much remains after processing, how much is lost during storage, and how much is released at the intended stage of application [199,200,201]. For this reason, encapsulation efficiency should be separated from retention and functional release. A complete assessment should distinguish at least five parameters: total loaded active compound, surface-associated active compound, active compound retained after drying, active compound retained after storage, and active compound released under simulated use conditions [37]. Methods based only on solvent extraction immediately after preparation may overestimate performance, especially when the active compound is located near the surface of particles or is weakly associated with the matrix. In spray-dried powders, the surface-oil or surface-fragrance fraction is particularly relevant because this portion is more exposed to evaporation, oxidation, and interactions with oxygen or humidity. In hydrogel beads, the apparent encapsulation efficiency may be high, but rapid swelling or ion exchange can cause burst release after contact with aqueous media. In films and coatings, the active compound may be retained during preparation but lost gradually through evaporation before use [57]. Thus, retention is a time-dependent and environment-dependent parameter. It is affected by particle size, shell thickness, matrix density, water activity, filler porosity, storage temperature, humidity, oxygen exposure, and the affinity between the active compound, polymer, and inorganic phase. Inorganic components can improve retention when they provide internal adsorption sites, tubular reservoirs, mesopores, or diffusion barriers [206,207,208]. However, strong adsorption may become a disadvantage if the active compound cannot be released effectively. Accordingly, the design of hybrid carriers should balance three requirements: efficient incorporation, protection during storage, and controlled release under application-relevant conditions. For quantitative comparability, the equation, denominator, mass basis, and units used for each performance metric should be stated explicitly. For the purposes of this review, encapsulation efficiency is expressed as EE (%) = (mass of payload retained in the carrier after preparation/mass of payload initially used) × 100; loading capacity as LC (%) = (mass of payload retained in the carrier/mass of the final loaded carrier) × 100; retention (%) = (payload remaining after a defined processing or storage interval/payload present at the selected reference time) × 100; and cumulative release (%) = (cumulative mass released at time t/initially encapsulated payload mass) × 100. When alternative definitions are used, the denominator and wet- or dry-mass basis should be clearly reported. Loading may additionally be expressed in mass units such as mg/g.

Release from hybrid polysaccharide systems may involve several concurrent or overlapping mechanisms, including diffusion through hydrated polymer networks, swelling-controlled transport, polymer-chain relaxation, matrix degradation, or surface erosion, changes in ionic crosslinking, and payload-matrix interactions [46,209,210]. In ionically crosslinked systems, ion exchange can reduce crosslink density, promote swelling and ultimately cause de-crosslinking or matrix disintegration [183,211]. Mineral-containing hybrids may additionally release payloads through desorption and diffusion from clay interlayers or mesoporous mineral fillers [198,212]. The relative contribution of these processes depends on payload polarity or charge, molecular size, volatility, particle size and geometry, network swelling and mesh size, filler porosity, and experimental conditions such as pH, ionic strength, temperature, hydrodynamics, and digestion conditions [2,5,10,15,28,209,213,214]. Hydrophilic actives are commonly released following water penetration and diffusion through swollen aqueous networks, whereas hydrophobic compounds generally require solubilization or partitioning from oil droplets, micelles, or other hydrophobic domains before transfer to the surrounding aqueous medium [184,215]. For volatile compounds, vapor pressure and gas-matrix partitioning introduce an additional evaporation or headspace-release pathway. Consequently, liquid-contact release measurements and air or headspace measurements represent different experimental endpoints and should not be considered directly interchangeable [216].

**Figure 4:**
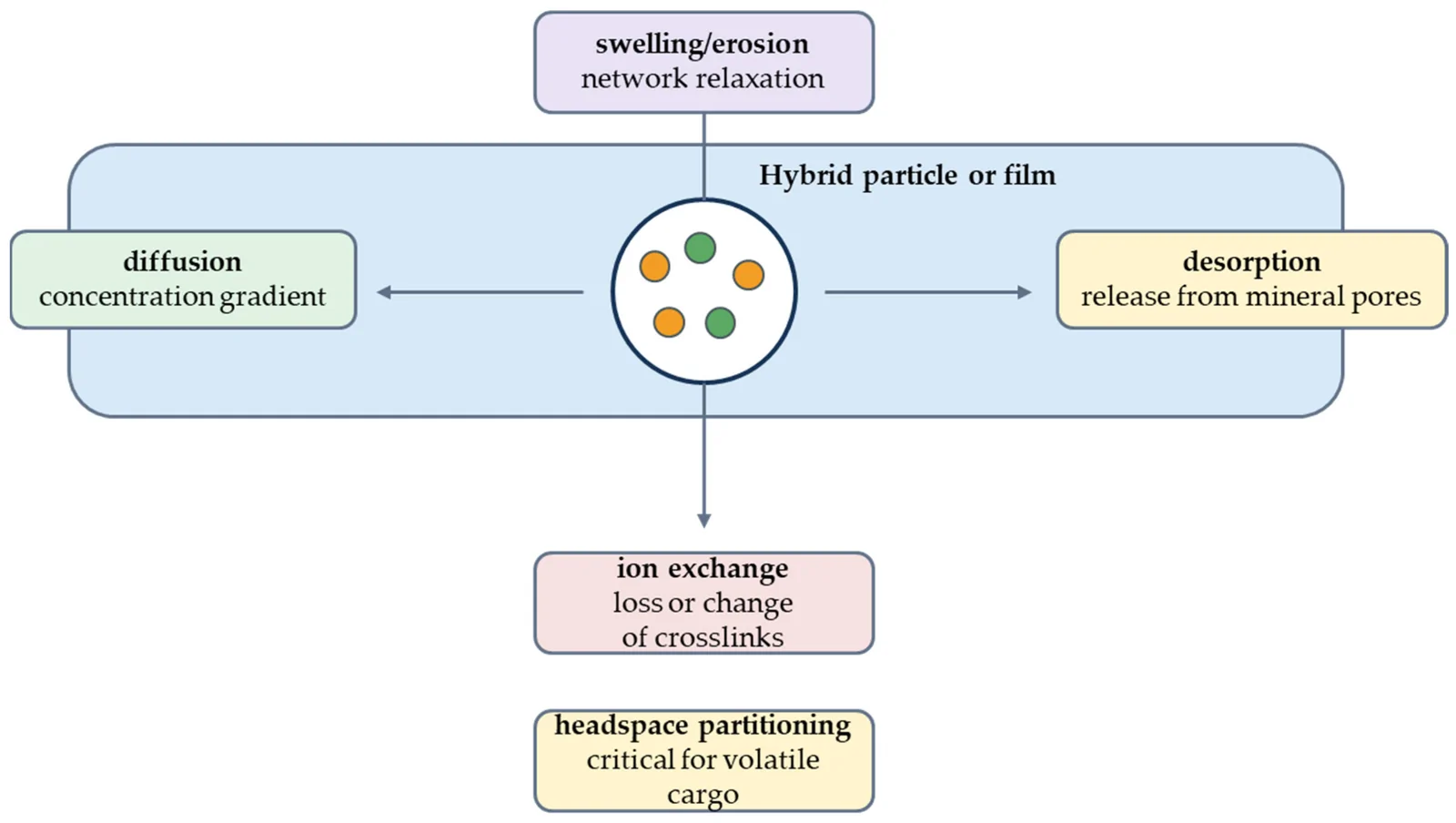
Schematic representation of the possible release mechanisms in polysaccharide-inorganic hybrids.

The main mechanisms involved in release from polysaccharide-inorganic hybrid carriers are summarized in Figure 4.

Pore architecture and carrier dimensions are additional structural parameters governing encapsulation and release because they determine payload accessibility, available interfacial area, and diffusion-path length. In alginate hydrogels, controlled variation in porosity has been shown to modify prolonged drug release, indicating that larger or more interconnected pores can facilitate hydration and molecular transport, whereas restricted pore networks can retard diffusion [193]. In hybrid systems, mesoporous inorganic phases provide a second level of confinement: increasing the mesoporous silica fraction in polysaccharide hydrogel beads enhanced sustained release and enabled bovine serum albumin delivery over 24 h [29]. For volatile compounds, mesoporous silica with a pore size of approximately 4.5 nm and a specific surface area of 1120 m^2^/g enabled high loading and retained approximately 50% of the encapsulated compounds after two months, illustrating how pore confinement and surface interactions can markedly reduce premature loss [198]. Carrier dimensions exert a complementary effect: alginate/halloysite hydrogel wires with diameters of approximately 0.19 and 0.47 mm required about 6 and 10 h, respectively, to release 50% of salicylic acid, compared with approximately 2 h for drug-loaded halloysite without the alginate matrix [44]. Thus, neither smaller pores nor smaller particles are universally advantageous. Release depends on the balance between pore accessibility, payload-surface interactions, surface-area-to-volume ratio, and diffusion distance. Consequently, excessive confinement may prolong release or hinder complete payload liberation.

**Figure 5:**
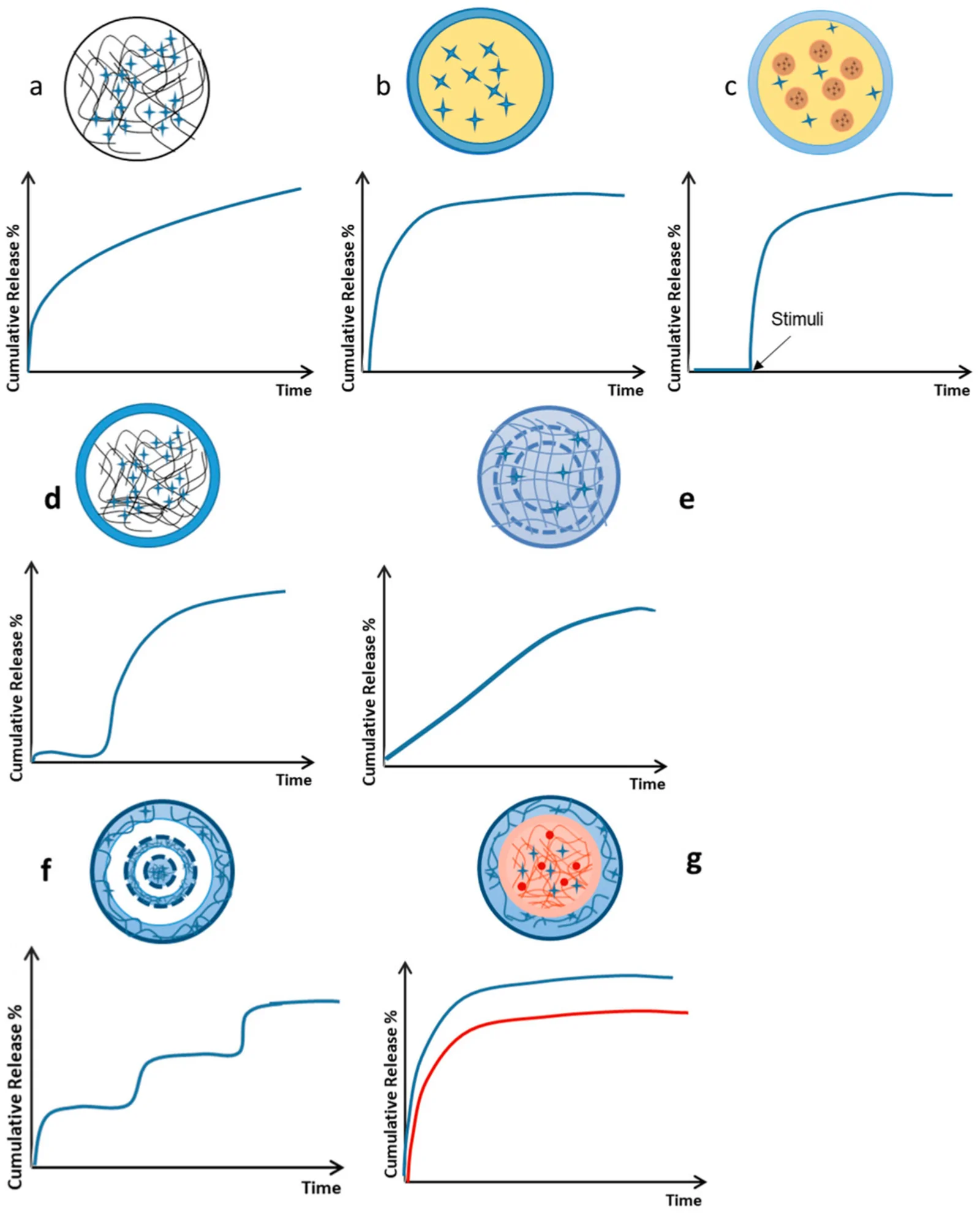
Schematic illustration of typical chitosan sub-microparticulate carriers and corresponding release profiles found in the literature. The shuriken-shaped blue marks and red dots stand for active ingredients loaded. (**a**) Monolithic sphere; (**b**) capsule with a liquid core; (**c**) nanoparticle-loaded capsule with a CS shell; (**d**) core-shell sphere with a CS matrix core; (**e**,**f**) multilayered CS hydrogel capsule; (**g**) core-shell sphere loading two drugs. Reprinted from [47] (under the terms of the open access Creative Commons CC BY license of MDPI publisher).

Furthermore, the drug release kinetics depends strongly on the particulate building materials, the properties of the encapsulated species, and the structural characteristics of the carriers, including particle shape, particle size, surface roughness, porosity, pore size, and shell thickness. The example is shown in Figure 5, which summarizes several representative controlled-release particulate chitosan-based carriers and their corresponding release profiles reported in the literature.

Mathematical models such as zero-order, first-order, Higuchi, Korsmeyer-Peppas, and Weibull equations are useful for comparing release profiles, but their applicability limits should be stated explicitly, as they should not be applied without physical interpretation. Zero- and first-order equations are primarily kinetic descriptors, whereas a Higuchi interpretation is most defensible for diffusion-dominated matrix release when the assumed geometry, approximately constant diffusivity, and sink or near-sink conditions are appropriate. The Korsmeyer-Peppas exponent is geometry-dependent and should be interpreted only within the fitting range for which the model assumptions remain valid; the Weibull equation is empirical and should not be treated as proof of a release mechanism [217,218,219]. A good mathematical fit does not necessarily prove a specific mechanism [217]. For example, a Higuchi-like profile may result from diffusion through a porous matrix, but it may also reflect a combination of swelling, desorption, and changing concentration gradients [210,218]. Similarly, the exponent in the Korsmeyer-Peppas equation can suggest diffusion- or relaxation-controlled release, but only if the tested system meets the assumptions of the model [219]. In polymer-inorganic hybrid materials, release mechanisms are often mixed because, swelling, desorption, ion exchange, partitioning, erosion, and diffusion may operate simultaneously [181,210,218,220]. Therefore, kinetic modelling should report carrier geometry, release-medium conditions, the fitted time or release fraction, and goodness-of-fit criteria, and should be interpreted together with mass balance, swelling, morphology, and payload-localization data.

For hydrophilic active compounds, release is often accelerated by matrix hydration, swelling, and dissolution. Alginate, pectin, starch, and some cellulose derivatives can absorb water rapidly, which facilitates diffusion but may also reduce long-term retention [180,183,209]. Additional coatings, denser crosslinking, or inorganic fillers can slow release by reducing water uptake or increasing diffusion path length [180,181]. In the case of proteins, enzymes, or probiotics, release assessment should include biological activity or viability, because chemical loading does not necessarily mean that the active compound remains functional after encapsulation and release [74,96,149,221,222].

For hydrophobic or volatile compounds, release may be governed less by matrix dissolution and more by partitioning. In emulsion-based carriers, hydrophobic actives first partition from dispersed oil droplets or other nonpolar domains into the surrounding aqueous or polymer phase and subsequently diffuse through the hydrated matrix. When an active compound is confined within an inorganic reservoir, such as a halloysite lumen or a mesoporous silica pore, release additionally requires desorption from the mineral surface before diffusion through the surrounding polysaccharide network. In coated capsules, the shell introduces an additional mass-transfer barrier, and its hydration and permeability influence the rate at which the active compound reaches the external phase. For sufficiently volatile flavors and fragrances, release may proceed one step further, with the compound partitioning from the carrier or surrounding phase into the gas phase (headspace). Subsequently, sensory performance depends on headspace concentration rather than solely on the amount recoverable by solvent extraction [223].

Beyond the release pathway itself, storage can further modify the retention and functional performance of encapsulated essential oils. Moser et al. monitored pink pepper essential oil in free and microencapsulated forms over 135 days and showed that microencapsulation reduced volatile losses and better-preserved antioxidant activity, while antimicrobial activity was retained in the encapsulated systems [224]. Similarly, Alarcón-Moyano et al. investigated alginate films containing microencapsulated lemongrass essential oil or citral over 28 days of storage, demonstrating that storage time and encapsulation architecture influence the retention and functional performance of volatile actives [169]. Consequently, release tests for volatile payloads should include information on humidity, temperature, headspace volume, agitation, sampling method, and calibration strategy phase [157,184].

The choice of analytical method should match the intended application. Solvent extraction is useful for determining total loading, but it does not describe release into air. Headspace analysis, gas chromatography, dynamic vapor sorption, or closed-system release tests are more appropriate for fragrances, flavors, and essential oils [156,223]. For antimicrobial essential oils, release studies should be combined with biological assays, because the concentration released may not directly correspond to antimicrobial efficacy after storage [224]. For nutraceuticals and phenolic compounds, antioxidant activity, degradation products, and pH-dependent stability should be considered [225]. For cosmetic active compounds, release should be evaluated in the final formulation or in media mimicking the formulation, because surfactants, oils, humectants, and emulsifiers can strongly modify partitioning and diffusion [226].

The relationship between active compound class and carrier design is summarized in Table 5. It emphasizes that different active compounds require different matrix-filler logic and different release testing strategies.

## 7. Hybrid Carriers for Volatile and Bioactive Compounds

### 7.1. Flavors, Fragrances, and Essential Oils

The use of polysaccharide-based organic-inorganic hybrid carriers for flavors, fragrances, and essential oils is of particular importance, as these valuable payloads are difficult to stabilize. Many of them are hydrophobic, volatile, oxygen-sensitive, and susceptible to thermal loss during processing [239]. Conventional polysaccharide gels can encapsulate emulsified oils, but highly hydrated networks often provide limited affinity for nonpolar molecules and may allow rapid diffusion or evaporation. Hybridization with inorganic reservoirs such as silica, mesoporous silica, halloysite, clays, or layered double hydroxides can improve retention by adding adsorption sites, internal cavities, tortuous pathways, or barrier effects, while the polysaccharide phase provides a processable, biobased, and often formulation-compatible shell. For payloads like flavors, fragrances, and essential oils carrier performance should be evaluated using criteria that go beyond initial encapsulation efficiency. Studies of flavor and essential-oil encapsulation indicate that overall performance is governed by the combined influence of the fabrication method, particle morphology, wall-material chemistry, storage conditions, and release environment rather than by any single formulation parameter [65,199,200,201]. These observations support the central argument of this review that volatile payloads require integrated assessment of retention, headspace behavior, chemical stability, and application-specific release, not only extraction-based loading values.

Recent polysaccharide-based studies reinforce the importance of mineral- or nano-reinforced matrices for volatile-compound retention and formulation stability. Moreover, application conditions determine carrier design. Quantitative comparisons demonstrate that the magnitude of the formulation effect depends strongly on carrier architecture and the intended application. For rosemary essential oil, incorporation of montmorillonite into calcium alginate increased encapsulation efficiency from 81% to 83% and loading capacity from 71% to 73%. The hybrid micro-capsules also increased DPPH-measured free-radical reduction from 10.0% to 12.8% and prolonged oil release compared with calcium alginate alone [235]. In a food-flavor system, wall-material selection produced an even larger difference: gelatin-based microcapsules for jasmine instant tea showed a polyphenol-based encapsulation efficiency of 74–79%, whereas maltodextrin-based particles reached only 37–41%, and their residual moisture contents were 3.23% and 4.98%, respectively [240]. Therefore, simplified aqueous release assays should be complemented by at least one test performed in a real or simulated formulation [37,199,241]. These results illustrate also that the benefit of carrier modification should be assessed using application-relevant performance parameters rather than inferred from material composition alone.

A broader comparison of representative polysaccharide-inorganic carriers for essential oils and related volatile compounds is provided in Table 6.

### 7.2. Nutraceuticals, Phenolics, and Antioxidants

Nutraceuticals, phenolic compounds, carotenoids, vitamins, and plant extracts require protection against oxidation, light, heat, and pH changes. For this group of compounds, chemical stability is often as important as release rate. Polysaccharide-based hybrid carriers can combine edible or acceptable wall materials with inorganic components that improve barrier properties, water resistance, or structural integrity. Alginate, pectin, starch, maltodextrin, and chitosan are frequently selected because of their compatibility with food and nutraceutical applications. At the product level, alginate-based systems have been applied in foods such as yogurt and ice cream, as well as in emulsion-type products including low-fat mayonnaise, and edible films or coatings for food preservation [9].

In hybrid formulations, inorganic phase can contribute adsorption domains, tortuous diffusion paths, or improved film strength [9,49,57,59,74,233]. It should be noted that a carrier that releases an antioxidant efficiently may still fail if the compound is degraded during drying or storage. For this reason, studies on phenolics and nutraceuticals should report residual activity, antioxidant capacity, or degradation products in addition to loading and release. The choice of wall material and process parameters is especially important in drying-based encapsulation, where thermal exposure, oxygen contact, and residual moisture can influence both retention and bioactivity [232,242,243].

### 7.3. Proteins, Probiotics, and Biomedical Actives

Proteins, enzymes, probiotics, and selected biomedical actives benefit from mild aqueous processing, which explains the frequent use of alginate-based gelation and alginate-chitosan coatings. For these biological payloads, the main challenge is not only incorporation into the carrier, but preservation of biological activity [2,137,149,238]. Hybridization can improve mechanical stability, reduce burst release or provide secondary interactions with biomolecules, as observed in LDH-containing alginate hydrogels, silica-polysaccharide hydrogel beads, and coated polyelectrolyte systems. Recent probiotic and bioactive-delivery studies further emphasize that survival, biological activity, and release behavior should be evaluated together rather than inferred only from encapsulation efficiency [40,96,138,244]. The fabrication route must be adapted to the active compound. Sol-gel chemistry, drying, mineralization, and exposure to organic solvents or extreme pH may reduce protein activity or cell viability. Therefore, biological payloads should be evaluated by activity or viability assays after encapsulation, after storage, and after release. For probiotics, simulated gastric and intestinal conditions are more meaningful than release in water alone [245]. For enzymes and proteins, activity retention should be reported together with release kinetics [246,247].

### 7.4. Cosmetic and Household Formulations

Cosmetic and household products expose hybrid carriers to formulation environments that are more complex than simple laboratory media. Surfactants, salts, preservatives, oils, fragrance blends, rheology modifiers, and mechanical shear can alter swelling, de-crosslinking, adsorption, and diffusion. Many polysaccharide-based hybrids are evaluated in water or buffer, whereas their target matrices are creams, gels, fabric conditioners, detergents, powders, coatings, or textile surfaces. This gap is particularly important for fragrance carriers and cosmetic active compounds, where storage stability, odor profile, visual appearance, and compatibility with the final formulation are as important as release kinetics [241].

A key recommendation for future work in this section is to include at least one real or simulated product test. For cosmetic active compounds, release should be evaluated in the presence of surfactants, oils, or humectants typical for the formulation [248]. For household fragrances, carriers should be tested under mechanical stress, high ionic strength, and surfactant-rich conditions. Such tests would improve the relevance of hybrid carrier studies and support translation from proof-of-concept particles to application-ready systems [249].

## 8. Applications and Sector-Specific Performance Criteria

The same polysaccharide-inorganic platform can be adapted to multiple sectors, but each application imposes different performance criteria. In food and nutraceutical systems, edible status, sensory neutrality, oxygen protection, moisture stability, and release during consumption are central [25]. In cosmetics, skin compatibility, visual appearance, odor profile, emulsion stability, and compatibility with preservatives or surfactants become critical [241,250]. In household products, mechanical robustness, fragrance retention, compatibility with detergents, and release under friction or hydration are more relevant than physiological release conditions [251]. Biomedical use requires cytocompatibility, sterility, degradation behavior, and well-defined release in physiological media [252,253]. Packaging applications demand film strength, oxygen, and water-vapor barrier properties, optical quality, migration assessment, and stability under humidity [58,59,60,61,87,254].

This diversity explains why a universal best carrier does not exist. Alginate is excellent for mild gelation and aqueous encapsulation, but it may be insufficient for hydrophobic fragrance retention unless it is combined with an adsorptive or barrier-forming inorganic reservoir. Chitosan is useful for pH response, antimicrobial activity, and polyelectrolyte complexation, but it may require acidic processing and may not be compatible with all neutral or alkaline formulations. Nanocellulose improves mechanical and barrier properties in films, but it does not automatically solve loading and retention problems for hydrophobic bioactives. Starch and maltodextrin are valuable for spray-dried powders, but they can be sensitive to humidity. The rational strategy is therefore to select the matrix-filler pair according to encapsulated compound polarity, volatility, required triggering factor, and target product environment. A design-oriented review should therefore avoid ranking materials in general terms. Instead, it should identify which material features are desirable for each application sector. In food powders, dry stability and sensory release may dominate. In cosmetic emulsions, compatibility with the formulation may be the limiting factor. In biomedical carriers, cytocompatibility and reproducible release are essential. In packaging, migration and barrier performance are decisive. Such sector-specific interpretation makes structure-property relationships more useful for future material design.

## 9. Scalability, Safety, and Sustainability

Application-ready hybrid carriers require more than promising laboratory release curves. Processing routes such as spray drying, extrusion, coating, and continuous gelation are more directly scalable than manual dripping or low-throughput batch preparation. However, scale-up can change droplet size, residence time, drying rate, residual moisture, filler distribution, and encapsulated-compound loss. Therefore, future studies should connect processing parameters with final carrier properties rather than reporting only optimized laboratory batches. Scalability is particularly relevant for flavors, fragrances, and nutraceutical powders because these applications often require kilogram- or ton-scale production, reproducible particle size, stable storage, and compatibility with existing industrial processes. Spray drying and extrusion are attractive in this respect, but they require careful control of feed viscosity, solid content, atomization, thermal exposure, and drying yield. For films and coatings, scale-up depends on casting or coating uniformity, drying energy, and substrate adhesion. For hydrogel beads, continuous gelation or controlled extrusion is more relevant than manual dropping.

Safety assessment becomes essential whenever inorganic phases are added. A polysaccharide matrix may be biodegradable or biocompatible, but the hybrid material can still release particles, ions, or residual precursors. This issue is especially important in food-contact, cosmetic, and biomedical applications. Migration tests, cytocompatibility, degradation, and environmental-fate studies, as well as filler-leaching assessments should be selected according to the intended sector rather than applied generically. For example, performance gains in polysaccharide packaging functionalized with inorganic nanoparticles must therefore be balanced with safety, migration, and regulatory considerations.

Sustainability should also be treated as a material property. The renewable origin of a polysaccharide does not automatically make the complete hybrid system sustainable. Extraction route, polymer grade, crosslinker choice, solvent use, drying energy, filler source, biodegradability, recyclability, end-of-life behavior, waste generation, and compatibility with existing production lines all influence environmental performance. A carrier made from a bio-based polymer but processed through energy-intensive drying or combined with a persistent inorganic nanofiller may have limited practical sustainability. In practical terms, the availability of natural polysaccharide matrices provides a favorable basis for sustainable carrier design, provided that the selected matrix-filler combination achieves the required retention and release performance without compromising whole-system sustainability [16,20,21,67,68]. Consequently, future studies should discuss sustainability at the level of the whole system, not only the organic matrix. Accordingly, formal life-cycle assessment should be prioritized in future studies to quantify these trade-offs and enable comparison among competing hybrid carrier designs.

## 10. Recommendations for Reporting in the Literature

A recurring weakness in the literature is insufficient comparability between hybrid carrier studies. Many papers report morphology, loading, and release, but omit variables that strongly affect interpretation, such as particle-size distribution, residual moisture, surface-associated active compound, filler dispersion, filler leaching, headspace volume, storage conditions, or release mass balance. For materials intended for volatile compounds, these omissions can change the meaning of the data. A slow apparent release may result from true diffusion control, but it may also reflect evaporation, degradation, adsorption on the vessel wall, or incomplete extraction.

The minimum set of descriptors needed for comparable studies is summarized in Table 7. These parameters should not be treated as optional details, since they connect formulation, processing, and material structure with the observed release profile. Reporting them systematically would make it easier to compare systems prepared by different fabrication routes, such as gelation, spray drying, extrusion, casting, or electrospinning.

For volatile payloads, the characterization checklist should be expanded to include headspace analysis, storage retention, and the surface-associated fraction of the active compound. For protein and probiotic systems, activity or viability should be reported in parallel with release. For materials containing inorganic nanophases, filler leaching, ion release, or particle migration should be assessed according to the intended application. These additions are necessary because high loading alone does not guarantee successful functional performance.

## 11. Current Challenges and Research Gaps

The remaining research gaps concern comparability and translation rather than the availability of candidate materials. Release protocols remain heterogeneous, particularly for volatile payloads, while long-term storage stability, filler distribution and retention, and validation in end-use formulations are still insufficiently reported. Simplified aqueous tests may not predict behavior in emulsions, detergents, packaging systems, or bio-logical fluids, and incomplete reporting of migration/leaching, process yield, particle-size distribution, and batch reproducibility limits safety and scale-up assessment. Future studies should therefore link the minimum descriptors summarized in Table 7 to application-relevant performance, using exposure-appropriate safety tests and realistic release media, so that changes in matrix-filler interactions, morphology, and processing can be related directly to reproducible functional outcomes.

## 12. Design Guidelines for Future Hybrid Carriers

A practical design workflow should begin with the payload and its dominant constraints, including polarity or volatility, oxidation sensitivity, required trigger, preservation of biological activity, mechanical demand, and production scale. The polysaccharide matrix, inorganic phase, and fabrication route should then be selected as an integrated system rather than optimized independently. Table 8 summarizes this payload-first decision framework by linking these requirements with matrix logic, suitable inorganic phases, processing routes, and the most relevant evaluation metrics.

The design logic in Table 8 emphasizes complementarity between the three components of the carrier: the polysaccharide provides processability and responsive chemistry, the inorganic phase should address the dominant limitation through adsorption, barrier formation, reinforcement, ion exchange, or stimulus response, and the fabrication route must preserve the payload while producing the required geometry at the intended scale. Accordingly, optimization should target a balanced matrix-filler-processing combination rather than maximize a single parameter such as encapsulation efficiency.

Nevertheless, food and cosmetic suitability should be assessed for the specific formulation rather than assigned broadly to an entire material class, since acceptability depends on material grade, loading, migration, exposure route, and intended application. Furthermore, direct food use should be distinguished from food-contact packaging because the relevant exposure and migration conditions differ.

## 13. Conclusions

Polysaccharide-based organic-inorganic hybrid carriers are best considered as coupled matrix-filler-processing systems rather than as biopolymers containing passive additives. Alginate is a useful reference matrix because of its mild aqueous gelation and versatility, whereas chitosan, cellulose/nanocellulose, starch/maltodextrin, pectin, carrageenan, and related polysaccharides provide complementary charge, film-forming, barrier, rheological, and processing characteristics that may be better suited to specific payloads and carrier formats. By critically organizing the literature published between 2016 and 2026 within a structure-property-application framework, this review evaluates how poly-saccharide chemistry, inorganic-phase characteristics, interfacial interactions, and fabrication methods determine encapsulation efficiency, payload stability, mechanical integrity, barrier properties, and release kinetics. It also compares major processing strategies, including ionic gelation, coacervation, casting, extrusion, spray drying, freeze-drying, emulsion-based methods, LbL assembly, and additive manufacturing, with particular attention to scalability and compatibility with sensitive payloads.

Performance of polysaccharide-based organic-inorganic hybrid carriers is governed by coordinated matching of matrix chemistry, inorganic-phase architecture, processing-induced morphology, and payload properties. Porous or tubular fillers such as mesoporous silica and halloysite are most useful when adsorption and diffusion control dominate. LDH and MOF phases are advantageous for ion-exchange or stimulus-responsive delivery. Hydroxyapatite is primarily relevant to bioactive and mechanically reinforced systems, whereas metal oxides or magnetic particles add antimicrobial, catalytic, or externally responsive functions. Processing determines how these functions are expressed by controlling carrier geometry, porosity, interfacial contact, and payload exposure to heat, solvents, or shear. The optimal carrier is therefore not necessarily the system with the highest loading or strongest interactions, but the one that balances payload retention during storage with controlled release under the intended conditions. For volatile compounds, this balance additionally requires consideration of gas-phase partitioning and oxidation, making functional retention more informative than encapsulation efficiency alone.

The principal design implication is that no universal best hybrid carrier exists. Thus, future progress should prioritize comparative, application-driven optimization of these coupled design variables rather than additional isolated proof-of-concept formulations.

## Figures and Tables

**Table 1:** Polysaccharide matrices and their roles as the carriers of active substance.

Matrix	Key Functional Groups	Main Carrier Formats	Strengths	Typical Limitations	References
Alginate	Carboxylates	Beads, hydrogels, films, and coatings	Mild gelation, aqueous processing, and high biocompatibility	Ion exchange, swelling, and weak mechanics	[1,2,3,4,5,6,9,10]
Chitosan	Amines and hydroxyls	Nanoparticles, coatings, hydrogels, and films	Cationic, antimicrobial, pH-responsive, polyelectrolyte complexation	pH-dependent solubility, sensory, and compatibility issues	[17,45,47,48,49,50,51,52,53,54,77]
Cellulose/nanocellulose	Hydroxyls	Films, aerogels, foams, fibers	Reinforcement, barrier improvement, and dimensional stability	Limited solubility and dispersion challenges	[63,64,65,73,78,79]
Starch/maltodextrin	Hydroxyls	Spray-dried powders, granules, and films	Food-grade, scalable, and inexpensive	Moisture sensitivity and rapid release	[21,69,80,81]
Pectin/carrageenan/gums	Carboxyl, sulfate or hydroxyl groups	Gels, films, and rheology modifiers	Edible, formulation-friendly, and viscosity control	Variability and humidity sensitivity	[82,83,84,85]

**Table 2:** Functional roles of inorganic phases in polysaccharide-based hybrid carriers.

Inorganic Phase	Main Function	Usefulness	Potential Risk or Limitation	References
Silica/mesoporous silica	Reinforcement, porosity, and reservoir effect	Hydrophilic and hydrophobic actives, enzymes, and controlled release	Aggregation, strong adsorption, and particle release	[28,29,30,31,32,33,55,91,92]
Halloysite/clays	Tubular reservoirs, tortuous diffusion, and barrier improvement	Essential oils, fragrances, antimicrobials, and packaging	Brittleness, sedimentation, and dispersion limits	[34,44,93,94,95]
Layered double hydroxides	Anion exchange, pH response, protein binding	Therapeutics, antimicrobials, and responsive release	Ion release and filler loading limits	[34,96,97]
Metal NPs and metal oxides	Antimicrobial, and photocatalytic or magnetic functions	Active films, wound materials, smart carriers	Toxicity and migration assessment	[24,58,61,63,73]
Hydroxyapatite	Bioactivity and mineral reinforcement	Bone-related carriers and tissue engineering	Limited relevance to volatile compounds	[98,99,100,101]
MOF/mineral hybrids	High surface area and stimulus response	Advanced drug and bioactive delivery	Water stability and safety questions	[89,90,102,103]

**Table 3:** Comparison of fabrication strategies for polysaccharide-based organic-inorganic hybrid carriers.

Strategy	Typical Product	Advantages	Key Descriptors to Report	Main Application Fit	References
Ionotropic gelation	Hydrogel beads, capsules	Mild, aqueous, compatible with labile active compounds	Polymer concentration, ion type, ion concentration, droplet size, and gelation time	Probiotics, enzymes, and hydrophilic active compounds	[1,2,40,136,137,138]
Polyelectrolyte complexation/LbL	Core-shell particles and coated fibers	pH response and reduced burst release	Layer sequence, zeta potential, coating thickness, pH	Drug delivery and responsive coatings	[17,45,46,47,51,139,140]
Sol-gel/in situ mineralization	Silica-integrated beads or hydrogels	Better interface and mineral distribution	Precursor ratio, pH, catalyst, aging, and washing	Silica-polysaccharide hybrids	[28,29,30,31,32,33,55,91,92]
Spray drying	Dry microparticles	Scalable and cost-effective	Feed composition, inlet/outlet temperature, yield, and residual moisture	Flavors, fragrances, and nutraceutical powders	[28,141,142]
Extrusion	Granules and beads	Scalable, simple, and suitable for food matrices	Nozzle, flow rate, drying conditions, particle size	Food and household products	[143,144]
Casting/electrospinning	Films, coatings, and fibers	High surface area or barrier layers	Solvent, blend ratio, humidity, thickness, and porosity	Packaging, textile, and cosmetic applications	[58,134,145,146]

**Table 4:** Structure-property descriptors relevant to polysaccharide-based organic-inorganic hybrid carriers.

Structural Level	Key Descriptor	Main Effect on Material Properties	Relevance for Encapsulation and Release	References
Molecular scale	Functional groups, charge density, crosslinking ion, degree of deacetylation or esterification	Defines ionic complexation, hydrogen bonding, coordination, and pH response	Determines matrix formation, encapsulated-compound affinity, and sensitivity to pH or ionic strength	[45,170,177,178]
Interface	Matrix-filler adhesion, surface chemistry, zeta potential, and electrostatic compatibility	Controls filler immobilization, interfacial continuity, and defect formation	Influences burst release, long-term retention, and structural stability	[28,44,175]
Colloidal scale	Filler size, shape, aspect ratio, and dispersion quality	Determines reinforcement, tortuosity, porosity, and aggregation risk	Controls diffusion pathways and accessibility of inorganic reservoirs	[160,173,179]
Network morphology	Pore size, network density, shell thickness, bead or film microstructure	Regulates swelling, permeability, and mechanical resistance	Determines release rate and protection of the encapsulated compound	[34,110,172,180,181]
Product geometry	Bead, capsule, powder, film, coating, fiber, or aerogel	Defines surface area, water uptake, and release behavior	Determines application-specific release behavior and storage stability	[1,145,156]
Environmental response	pH, humidity, ionic strength, surfactants, and temperature	Modifies swelling, plasticization, ion exchange, and matrix relaxation	Critical for real formulations, storage conditions, and trigger-dependent release	[164,182,183,184]
Encapsulated compound interaction	Hydrophilicity, volatility, oxidation sensitivity, and adsorption affinity	Affects encapsulation efficiency, surface localization, and retention	Particularly important for flavors, fragrances, essential oils, and labile bioactives	[157,185]

**Table 5:** Active compound classes and design implications for hybrid polysaccharide carriers.

Class of Active Compound	Key Challenge	Preferred Matrix/Filler	Release Test Requirement	References
Flavors and fragrances	Volatility, oxidation, and sensory release	Polysaccharide barrier plus silica, clay, or halloysite reservoir	Closed-system or headspace analysis; storage retention	[157,174,227]
Essential oils	Poor water solubility and antimicrobial activity loss	Emulsion-based wall plus clay, silica reinforcement	Oil phase partitioning, antimicrobial assay and retention	[65,75,77,95,150,165,166,167,199,200,201,202,203,224,228,229,230,231]
Phenolics and nutraceuticals	Oxidation and pH degradation	Alginate, pectin, starch, or chitosan with barrier or antioxidant fillers	pH-dependent release and antioxidant activity	[49,59,74,232,233]
Proteins/probiotics	Activity preservation and oxygen sensitivity	Mild alginate gelation, coated shells, LDH, or silica reinforcement	Viability/activity and release under gastric/intestinal conditions	[40,96,137,138]
Cosmetic actives	Formulation compatibility and storage stability	Hybrid films, beads, or microcapsules compatible with surfactants	Release in final formulation, not only water	[234,235,236]
Biomedically or environmentally relevant substances	Triggered release and adsorption	Alginate/chitosan/silica, MOF or LDH hybrids	Medium composition, pH, ions, and cytocompatibility	[2,4,29,42,45,46,54,55,78,90,97,149,237,238]

**Table 6:** Comparative performance of representative polysaccharide–inorganic carriers for essential oils and related volatile compounds.

Matrix Composition	Inorganic Filler/Reservoir	Active Compound	Inorganic Phase Function	Encapsulation/Loading	Release/Stability	Main Limitation	Application	Reference
Calcium alginate	Montmorillonite (MTN)	Rosemary essential oil	Adsorption and release retardation	EE: 81% → 83%; Loading: 71% → 73%	Prolonged release after MTN addition	Relatively modest increase in EE and loading	Controlled essential oil delivery	[235]
Calcium alginate microbeads	HNTs, LDHs, HNT@LDH	Grapefruit seed oil	Reservoir effect, confinement and tortuosity-controlled release	Oil to filler ratio up to 50:50 *w*/*w*; conventional EE not reported	Filler-dependent sustained release	Conventional EE not reported	Natural antimicrobial delivery	[34]
Chitosan/alginate coating	Modified halloysite nanotubes	Carvacrol	Physical barrier, controlled release and protection against external stress	Loading efficiency 26.33%	94.90% retained after 6 h of UV exposure; 83.61% retained after heat exposure	Not explicitly identified in the study	Food preservation	[86]
Sodium alginate film	Etched halloysite nanotubes	Cinnamaldehyde	Tubular carrier and diffusion retardation	Conventional EE not reported	Release into fatty-food simulant delayed by 144 h	Conventional EE not reported	Antimicrobial packaging	[174]

EE—encapsulation efficiency; HNT—halloysite nanotube; LDH—layered double hydroxide. Where conventional encapsulation efficiency was not reported in the original study, the available loading metric is provided without conversion to an EE value.

**Table 7:** Minimum characterization checklist for comparable hybrid carrier studies.

Descriptor	Why It Matters	Recommended Reporting
Composition	Connects formulation with performance	Polymer type, molecular weight or grade, filler type, filler loading, crosslinker, and encapsulated-compound loading.
Morphology	Controls diffusion and release geometry	Particle size distribution, shell thickness, pore structure, film thickness, and SEM/optical images.
Interfacial interactions	Determines stability and release	FTIR, zeta potential, XRD, thermal analysis, rheology, or swelling data.
Encapsulation and retention	Separates loading from storage stability	Encapsulation efficiency, loading capacity, surface-associated encapsulated compound, loss during drying, and loss after storage.
Release conditions	Controls comparability	Medium, pH, ionic strength, humidity, temperature, agitation, headspace, and sampling method.
Safety and sustainability	Needed for application	Migration, cytocompatibility or toxicity, filler leaching, biodegradation, and process scalability.

**Table 8:** Design guidelines connecting payload requirements with matrix-filler-processing choices.

Encapsulated Compound Requirement	Recommended Matrix Logic	Useful Inorganic Phase	Promising Processing Route	Main Evaluation Metric
High volatility	Hydrophilic shell plus adsorptive reservoir	Halloysite, silica, and LDH	Spray drying, extrusion, and coating	Retention after storage and release into headspace
Oxidation sensitivity	Barrier-forming polymer and low-oxygen processing	Silica and clays	Emulsification plus drying or film casting	Chemical stability and antioxidant/sensory retention
pH-triggered release	Alginate-chitosan complex or pH-responsive coating	LDH, MOF, and clay	LbL deposition or coated beads	Release at target pH and minimized burst
High mechanical demand	Nanocellulose-reinforced matrix or film	Clay, silica, and HAp	Casting, extrusion, and aerogel formation	Modulus, fracture behavior, and swelling
Food/cosmetic compatibility	Application-appropriate polysaccharide grade	Application-appropriate inorganic phase (specified grade and justified loading)	Spray drying, extrusion, and emulsion casting	Migration, sensory compatibility, and stability
Scalable production	Low-viscosity feed and robust drying	Dispersible filler with stable suspension	Spray drying, continuous gelation or extrusion	Yield, throughput, and batch reproducibility

## Data Availability

No new data were created or analyzed in this study. Data sharing is not applicable to this article.

## Supplementary Materials

The following supporting information can be downloaded at: https://www.mdpi.com/article/10.3390/polym18172047/s1, Table S1: Search concept blocks and representative terms; Table S2: Inclusion and exclusion criteria.
