# Supplementary material for: Polysaccharide-Based Organic-Inorganic Hybrid Carriers with Alginate as a Reference Matrix: Structure-Property Relationships and Emerging Applications in Encapsulation and Controlled Release
Source: Polymers (Basel). 2026 Aug 23;18(17):2047. doi: 10.3390/polym18172047 (PMC13568166; doi:10.3390/polym18172047)
Supplement: Supplementary file 1 [file polymers-18-02047-s001.zip › polymers-4500355-supplementary.pdf]

Supplementary Materials

Polysaccharide-Based Organic-Inorganic Hybrid Carriers with Alginate as a Reference Matrix: Structure-Property Relationships and Emerging Applications in Encapsulation and Controlled Release

Agata Wawrzyńczak *, Agnieszka Kłosowska and Agnieszka Feliczak-Guzik *

Department of Applied Chemistry, Faculty of Chemistry, Adam Mickiewicz University in Poznań, Uniwersytetu Poznańskiego 8, 61-614 Poznań, Poland
* Correspondence: agata.wawrzynczak@amu.edu.pl (A.W.); agnieszka.feliczak-guzik@amu.edu.pl (A.F.-G.)

Table S1. Search concept blocks and representative terms.

Concept block	Purpose	Terms combined with OR
A. Polysaccharide matrix	Identify the organic polymer phase	polysaccharide*; alginate; chitosan; cellulose; nanocellulose; cellulose nanofibril*; cellulose nanocrystal*; starch; maltodextrin; pectin; carrageenan; xanthan; pullulan; dextran; gum arabic; gellan gum
B. Inorganic phase/hybrid architecture	Identify a retained mineral, oxide, porous, layered, or magnetic component	organic-inorganic hybrid*; nanocomposite*; composite*; silica; mesoporous silica; clay*; halloysite; layered double hydroxide*; LDH; metal oxide*; zinc oxide; titanium dioxide; iron oxide; magnetite; maghemite; magnetic nanoparticle*; hydroxyapatite; calcium phosphate*; metal-organic framework*; MOF*
C. Encapsulation/release function	Restrict retrieval to carrier-relevant studies	encapsulat*; microencapsulat*; nanoencapsulat*; controlled release; sustained release; triggered release; delivery system*; carrier*; retention; payload*
D. Priority payloads (supplementary layer)	Increase sensitivity for volatile and bioactive applications	volatile*; flavor*; fragrance*; aroma*; essential oil*; cosmetic active*; nutraceutical*; bioactive*; antioxidant*; antimicrobial*

Table S2. Inclusion and exclusion criteria.

Domain	Inclusion criteria	Exclusion criteria
Material system	A natural or naturally derived polysaccharide is a structural matrix or co-matrix; a discrete inorganic phase is incorporated, grown in situ, deposited, or assembled and retained in the final hybrid.	Purely synthetic polymer systems; polysaccharide used only as a minor dispersant; soluble crosslinking ions without a retained inorganic phase; mixtures in which the phases do not jointly determine material properties.
Function and relevance	Encapsulation, loading, retention, protection, transport, or controlled release of an active compound; or a directly relevant structure-property study that informs carrier design.	Studies limited to pollutant adsorption/removal, catalysis, sensing, or mechanical reinforcement with no credible link to encapsulation or controlled-release design.
Payload	Volatile compounds, flavors, fragrances, essential oils, cosmetic actives, nutraceuticals, antioxidants, antimicrobials, drugs, dyes, proteins, or model molecules used to clarify release mechanisms.	No payload and no carrier-relevant structure-property evidence; payload identity or loading procedure not described sufficiently for interpretation.
Publication period	Core evidence published from 1 January 2016 to 1 July 2026 search date.	Core studies outside the date window unless explicitly classified as seminal contextual literature.
Publication type and language	Peer-reviewed original articles in English; peer-reviewed reviews used for background and citation chasing.	Conference abstracts without full papers, editorials, corrections, patents, theses, book chapters, non-peer-reviewed reports, and articles without accessible full text.
Minimum information	Material composition and preparation are sufficiently described; at least one relevant characterization, encapsulation, retention, stability, or release outcome is reported.	Insufficient methodological detail to establish matrix, inorganic phase, fabrication route, or outcome definition.
